# Exploring the cost-effectiveness of high versus low perioperative fraction of inspired oxygen in the prevention of surgical site infections among abdominal surgery patients in three low- and middle-income countries

**DOI:** 10.1016/j.bjao.2023.100207

**Published:** 2023-07-15

**Authors:** NIHR Global Health Research Unit on Global Surgery Collaborators, Bruce M. Biccard, Denton Smith, Shrikant Peters, Adam Boutall, Graeme Wilson, Ettienne Coetzee, Margot Flint, Simphiwe Gumede, Shreya Rayamajhi, Sharon Bannister, Nonkululo Daniel, Maria Fourtounas, Rachel Moore, Nnosa Sentholang, Osaheni Osayomwanbo, Aghadi Ifeanyi kene, Saidu Yusuf Yakubu, Amos Chukwu, Musliu Tolani, Yakubu Momohsani Adinoyi, Abdulrahman Aliyu, Dalhat Salahu, Isa kabir, Ibrahim Salisu, Tinuola Adigun, Anthony Adenekan, Emmanuel Williams, Pradeep Kumar Bhatia, Ramkaran Chaudhary, Nikhil Kothari, Sanjeev Misra, Puneet Pareek, Dharma Ram Poonia, Kirti Kumar Rathod, Mahaveer Singh Rodha, Naveen Sharma, Nivedita Sharma, Subhash Chandra Soni, Vaibhav Kumar Varshney, Jeewan Ram Vishnoi, Satya Shree Balija, Anuj Goyal, Farhanul Hudda, Manoji Joshva, Rajkumar Kottayasamy Seenivasagam, Shafiq Shajahan, Sameer Sharma, Sunil Kumar Singh, Praveen Talwar, Debendra Kumar Tripathi, Bhatt, Swati Daniel, Jyoti Dhiman, Christina George, Dhruva N. Ghosh, Sunita Goyal, Priyanka Hans, Parvez D. Haque, Deepak Jain, Harsharan Kaur, Karan Kumar, Amit Mahajan, Vishal Michael, Reuben Rajappa, Arti Rajkumar, Atul Suroy, Ravinder Singh Thind, Sreejith K. Veetil, Alisha Manisha Aggarwal, Parth Dhamija, Gurleen Kaur Garry, Himani Gupta, Ruchi Jakhar, Ashwani Kumar, Kshitij Kumar, Parmod Kumar, Gurtaj Singh, Sona Chowdhury, Neha Desai, Jyotsna Goswami, Sonia Mathai, Viplab Patro

**Affiliations:** a Groote Schuur Hospital, South Africa; b Chris Hani Baragwaneth Hospital, South Africa; c University of Benin Teaching Hospital, Nigeria; d Barau Dikko University Teaching Hospital, Nigeria; e Ahmadu Bello University Teaching Hospital Zaria, Nigeria; f Federal Medical Centre Birnin Kebbi, Nigeria; g Uduth Sokoto Hospital, Nigeria; h Aminu Kano Teaching Hospital, Nigeria; i Federal Medical Centre Gusau, Nigeria; j Federal Medical Centre Katsina, Nigeria; k University College Hospital Ibadan, Nigeria; l Obafemi Awolowo University Teaching Hospital, Nigeria; m NIHR Global Health Research Unit on Global Surgery, Nigeria; n All India Institute of Medical Science, Jodhpur, India; o All India Institute of Medical Science, Rishikesh, India; p Christian Medical College & Hospital, Ludhiana, India; q Government Medical College and Rajindra Hospital Patiala, India; r Tata Medical College, Kolkata, India

**Keywords:** abdominal surgery, cost-effectiveness analysis, global surgery, high fraction of inspired oxygen, low-and middle-income countries, surgical site infection

## Abstract

**Background:**

This study assessed the potential cost-effectiveness of high (80–100%) *vs* low (21–35%) fraction of inspired oxygen (FiO_2_) at preventing surgical site infections (SSIs) after abdominal surgery in Nigeria, India, and South Africa.

**Methods:**

Decision-analytic models were constructed using best available evidence sourced from unbundled data of an ongoing pilot trial assessing the effectiveness of high FiO_2_, published literature, and a cost survey in Nigeria, India, and South Africa. Effectiveness was measured as percentage of SSIs at 30 days after surgery, a healthcare perspective was adopted, and costs were reported in US dollars ($).

**Results:**

High FiO_2_ may be cost-effective (cheaper and effective). In Nigeria, the average cost for high FiO_2_ was $216 compared with $222 for low FiO_2_ leading to a −$6 (95% confidence interval [CI]: −$13 to −$1) difference in costs. In India, the average cost for high FiO_2_ was $184 compared with $195 for low FiO_2_ leading to a −$11 (95% CI: −$15 to −$6) difference in costs. In South Africa, the average cost for high FiO_2_ was $1164 compared with $1257 for low FiO_2_ leading to a −$93 (95% CI: −$132 to −$65) difference in costs. The high FiO_2_ arm had few SSIs, 7.33% compared with 8.38% for low FiO_2,_ leading to a −1.05 (95% CI: −1.14 to −0.90) percentage point reduction in SSIs.

**Conclusion:**

High FiO_2_ could be cost-effective at preventing SSIs in the three countries but further data from large clinical trials are required to confirm this.

Surgical site infections (SSI) are one of the most common adverse outcomes after abdominal surgery with incidence rates between 3.0% and 58.2%.^1^ The SSI rates are greater in low-income countries (23.2%) and middle-income countries (14%) compared with high-income countries (HICs) (9.4%).^2^ The prevention of SSIs is particularly important in low- and middle-income countries (LMICs), where the consequences can be particularly damaging to patients and health systems.^3^ The scarcity of perioperative oxygen in LMICs make consistent delivery of medical oxygen difficult or even impossible and the high demand for oxygen supplementation during the COVID-19 pandemic has further increased the scarcity of this healthcare resource.^4^^,^^5^

The World Health Organization (WHO) recommends administering an inspired fraction of oxygen (FiO_2_) of 80% during the perioperative period to adults undergoing general anaesthesia requiring tracheal intubation in order to prevent SSIs.^4^ The recommendation was made based on a consensus of a panel of experts and evidence from a meta-analysis of clinical trials which found that 80% perioperative FiO_2_ reduces SSIs compared with standard (30–35%) perioperative FiO_2_.^4^ The retraction of some RCTs that were initially included in the meta-analysis highlighted the challenges of the quality of this evidence base.^6^ The WHO have updated the guidelines based on a more recent meta-analysis (but have not changed the guidelines). However, a recent meta-analysis did not show clinical efficacy for 80% perioperative FiO_2_ at reducing SSIs compared with standard perioperative FiO_2_ (odds ratio 0.89, 95% confidence interval [CI]: 0.73–1.07) and there are ongoing debates on the safety of 80% perioperative FiO_2_.^7^^,^^8^

*The PErioperative respiratory care and outcomes for patieNts Undergoing hIgh risk abdomiNal surgery* (PENGUIN) trial is a randomised controlled trial (RCT) planned by the National Institute for Health and Care Research (NIHR) Global Health Research Unit on Global Surgery (Global Surgery Unit). The trial will assess the effectiveness of preoperative mouthwash and high perioperative FiO_2_ at preventing postoperative pneumonia and SSIs, respectively, among abdominal surgery patients in LMICs (NCT04256798).^9^ An internal PENGUIN pilot trial was ongoing, at the time of preparing this manuscript, to assess feasibility of randomisation, patient follow-up, and adherence to trial protocols.

Decision-analytic modelling is a systematic assessment under conditions of uncertainty, using mathematical relationships to estimate and compare possible costs and outcomes of interventions.^10^ The current study aimed at assessing the potential cost-effectiveness of high (80–100%) perioperative FiO_2_ (high FiO_2_) *vs* low (21–35%) perioperative FiO_2_ (low FiO_2_) at preventing SSIs among patients undergoing abdominal surgery in Nigeria, India, and South Africa using modelling.

## Methods

### Ethics statement

This model-based cost-effectiveness analysis (CEA) used unbundled data from the PENGUIN pilot trial collected from elective or emergency abdominal surgery patients aged ≥10 yr in India and South Africa (Supplementary Appendix S2) and data from the published literature. The PENGUIN pilot data were collected in line with standards set by the Helsinki Declaration. Ethical approval for the PENGUIN trial, including the pilot, was obtained from the University of Birmingham Science, Technology, Engineering and Mathematics Ethical Review Committee (Ethics number: ERN_19-1376). In India, ethical approval was granted by the Health Ministry Screening Committee and the trial was registered on the Clinical Trials Registry of India (CTRI/2020/08/027348). In South Africa, ethical approval was granted by University of Cape Town Health Research Ethics Committee (HREC 132/2020). In both countries, patients or guardians (for minors) provided written consent before being recruited into the trial.

### Economic evaluation

This CEA compared high and low FiO_2_ in terms of costs and outcomes where the costs were expressed in monetary units and outcomes were measured as SSIs.^11^ CEA results were reported using incremental cost-effectiveness ratio (ICER), that is the ratio of difference in expected costs to difference in SSIs. The ICER was compared with a cost-effectiveness threshold (CET), and when the ICER was below the CET the intervention was considered to be cost-effective.^10^ When high FiO_2_ was cheaper and effective compared with low FiO_2,_ it was considered to be ‘dominant’ (cost-effective) and when high FiO_2_ was expensive and less effective it was considered ‘dominated’ (not cost-effective).^10^^,^^11^

### Study perspective

A perspective defines the sectors from which costs and outcomes of an economic evaluation are included.^11^ This study used the healthcare perspective and quantified only costs and outcomes to the healthcare sector.^10^^,^^11^

### Type of model

**Fig 1:**
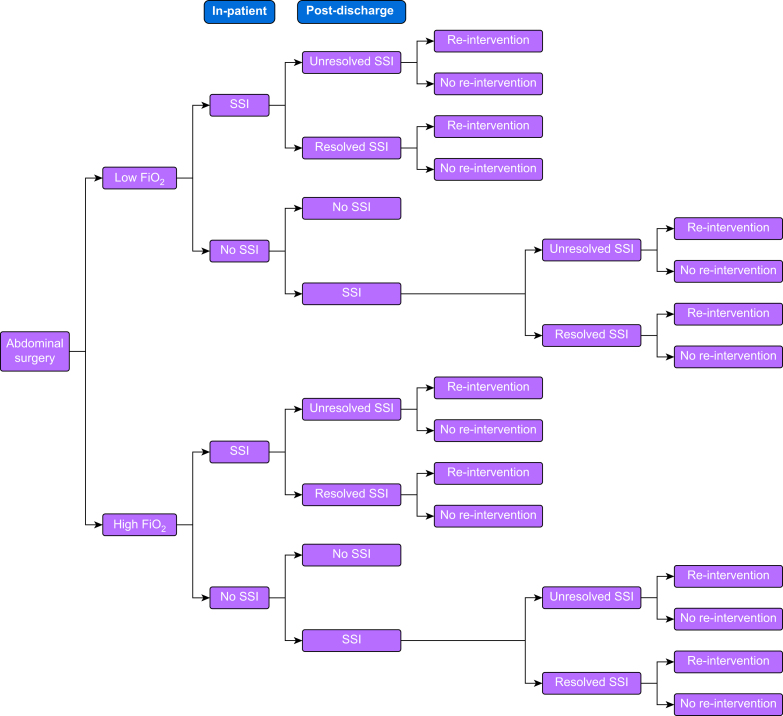
Patient pathways. FiO_2_, perioperative fraction of inspired oxygen; SSI, surgical site infection.

A decision tree was built because it is appropriate for modelling non-recursive conditions with a short timeframe.^10^^,^^12^ The tree depicted possible patient pathways of the PENGUIN trial.^9^ The decision was whether to administer high or low FiO_2_ and patients in both arms were anticipated to follow identical postoperative pathways (Fig 1). Some patients had in-patient SSIs, of which some resolved before hospital discharge. After discharge, a proportion of patients with resolved or unresolved SSIs needed re-intervention (either surgery or an interventional radiology procedure). Some patients developed an SSI after hospital discharge and a proportion of these SSIs resolved without the need for re-intervention. A proportion of patients with resolved or unresolved post-discharge SSIs needed re-intervention.

### Model timeframe

The model timeframe is the duration for which costs and outcomes are measured.^11^ The timeframe was the first 30 days after surgery in line with the US Centers for Disease Control and Prevention (CDC) criteria for superficial or deep SSI (Supplementary Appendix S2).^13^

### Effect size calculation

The model was informed by an updated meta-analysis on the effectiveness of high *vs* low FiO_2_ at preventing SSIs among patients undergoing surgery.^7^ Noting the conflicting evidence and the low quality of trials that had a positive effect in the meta-analysis, it was estimated that high FiO_2_ compared with low FiO_2_ had a 12.5% (CI: 5.2%–19.7%) relative risk reduction (RRR) with respect to SSIs. This was the minimum important clinical difference (MID) set in the PENGUIN trial.^9^

### Model probabilities

Probabilities reflect the expected likelihood of patients passing through model pathways.^10^ Probabilities for both arms were calculated from patient events using unbundled data (combined data for high and low FiO_2_) from the ongoing PENGUIN pilot trial. The data were collected and aggregated using the REDCap online data collection tool hosted at the University of Birmingham.^14^ The probabilities were assumed to be equal between the two arms, apart from the probability of in-patient SSI in the high FiO_2_ arm, and this was estimated by reducing the probability of in-patient SSI in the low FiO_2_ arm by 12.5% RRR (Table 1).

**Table 1:** Probabilities of patients passing through the pathways of the model. The probabilities between the two arms were assumed to be equal and were estimated from unbundled data, combined data of 585 high and low FiO_2_ patients (558 from India and 27 from South Africa) participating in the PENGUIN pilot study. FiO_2_, perioperative fraction of inspired oxygen; sd, standard deviation; SSI, surgical site infection. ∗Re-intervention after in-patient SSI. ^†^SSI occurring after the patient was discharged from the hospital. ^‡^SSI that occurred after hospital discharge and did not resolve. ^¶^Unresolved post-discharge SSI that needed re-intervention. ^§^However, the probability of in-patient SSI in the high FiO_2_ arm was estimated by reducing the probability of in-patient SSI in the low FiO_2_ arm by 12.5% relative risk reduction.

Variable	Baseline probability (sd)	Distribution	Source
**Low FiO**_**2**_			
In-patient SSI	0.08 (0.01)	Beta	PENGUIN pilot trial^9^
Unresolved in-patient SSI	0.53 (0.08)	Beta	PENGUIN pilot trial^9^
Re-intervention∗	0.50 (0.11)	Beta	PENGUIN pilot trial^9^
Post-discharge SSI^†^	0.02 (0.01)	Beta	PENGUIN pilot trial^9^
Unresolved post-discharge SSI^‡^	0.13 (0.12)	Beta	PENGUIN pilot trial^9^
Post-discharge re-intervention^¶^	0.002 (0.002)	Beta	PENGUIN pilot trial^9^
**High FiO**_**2**_			
In-patient SSI	0.07 (0.01)	Beta	PENGUIN pilot trial^9§^
Unresolved in-patient SSI	0.53 (0.08)	Beta	PENGUIN pilot trial^9^
Re-intervention∗	0.50 (0.11)	Beta	PENGUIN pilot trial^9^
Post-discharge SSI^†^	0.02 (0.01)	Beta	PENGUIN pilot trial^9^
Unresolved post-discharge SSI^†^	0.13 (0.12)	Beta	PENGUIN pilot trial^9^
Post-discharge re-intervention^¶^	0.002 (0.002)	Beta	PENGUIN pilot trial^9^

### Assumptions

The following assumptions were made. (1) Probabilities were consistent across health systems in the study because complication rates are similar in countries at similar levels of development.^15^^,^^16^ (2) In the base case, the median values for concentration rates for high and low FiO_2_ (90% and 28%, respectively) were used.

### Resource use and unit costs

Direct healthcare costs associated with perioperative oxygen and SSI treatment among abdominal surgery patients were estimated (Table 2). Resource use data were sourced from hospitals that are part of the Global Surgery Unit in Nigeria, India, and South Africa using a resource use questionnaire administered via REDCap (Supplementary Appendix S2).^14^ In Nigeria and India, unit costs were collected from the questionnaire whereas in South Africa, unit costs were sourced from the 2020 uniform patient fees schedule for the provincial and national public sector, because these are the actual billing costs to patients by the State.^17^ Discharge medication, purchased medication, and healthcare revisit costs in India and South Africa were collected from the PENGUIN pilot trial.^9^ Because PENGUIN pilot trial data from Nigeria were not available at the time of the analysis, healthcare revisit and purchased medication costs in Nigeria were imputed using the market-basket approach (Supplementary Appendix S2).^18^ Discharge medication costs from Nigeria were sourced from one centre participating in an international, multicentre trial, assessing the use of alcoholic chlorhexidine for skin cleaning and non-coated suture for wound closure at reducing SSIs in LMICs (FALCON).^19^ Costs in Nigerian Naira, Indian Rupee, and South African Rand were converted to 10.13039/501100004477US dollars ($) and then inflated to 2020 values (Supplementary Tables S6–S8).^20^^,^^21^

**Table 2:** Length of hospital stay. The LoS were sourced from clinical trials data, and it was assumed that LoS are identical between the two arms in the current model. LoS, Length of hospital stay; sd, standard deviation.

Variable	Mean LoS in days (sd)	Distribution	Source
SSI	16 (6)	Fixed	PENGUIN pilot trial^9^
No SSI	8 (4)	Fixed	PENGUIN pilot trial^9^
Re-intervention	18 (17)	Fixed	GlobalSurg Collaborative^16^

### Length of hospital stay

Length of stay data were sourced from the PENGUIN pilot study and GlobalSurg-2 datasets. The GlobalSurg-2 data included 6130 patients from LMICs that had an elective or emergency gastrointestinal resection.^9^,^16^ The length of stay was assumed to be identical between high and low FiO_2_ arms but different for SSI, no SSI, and reintervention (Table 2). Mean and standard deviation were used as there are more appropriate measures for costing studies even when the data are right skewed.^22^

### Outcome measure

The outcome measure was SSI at 30 days after surgery sourced from the unbundled PENGUIN pilot trial data (Supplementary Table S2). The data were aggregated to estimate the volume of SSIs for the unbundled data after a recommendation that summary statistics should be used in an economic evaluation if an analyst has access to primary data.^22^^,^^23^ The incidence of SSIs was converted to percentages and the arm with a lower SSI percentage was considered to be cost-effective compared to the other arm. In-patient SSI incidence in the intervention arm was estimated by reducing the incidence of the unbundled data by 12.5% RRR. Discounting of costs and outcomes was not done because of the short timeframe of the model.^10^^,^^11^

### Statistical analysis

The analysis was conducted in Microsoft Excel 2016 (Microsoft Corporation, Redmond, WA, USA). A roll-back method was used to estimate the expected costs and outcomes for both arms and a secondary analysis included purchased medication and travel costs.^10^ The ICER was calculated by dividing the difference in costs by the difference in SSIs and the results were reported as cost per percentage point of SSI reduced.

### Sensitivity analysis

Deterministic sensitivity analysis (DSA) was conducted to test the sensitivity of the base case results to changes in specific input parameters by changing a value of an input parameter while keeping the rest of the parameters constant. A parameter was considered sensitive if a change in the parameter's value changed the base case cost-effectiveness results (Supplementary Appendix S2).^11^

Probabilistic sensitivity analysis (PSA) is a technique for presenting uncertainty surrounding simultaneous changes of multiple input parameters where random values of the parameters are repeatedly drawn to re-estimate the difference in costs and outcomes based on parameter distributions.^10^ Probabilities were assigned a beta distribution and costs were assigned a gamma distribution (Supplementary Appendix S2). The PSA was run for 10 000 Monte-Carlo simulations and the results were presented using cost-effectiveness plane and cost-effectiveness acceptability curves (CEACs) at three times gross domestic product (GDP) per capita CET*.*^11^^,^^24^

## Results

### Base case results

Based on the current evidence on clinical effectiveness of FiO_2_ and the MID of 12.5% RRR, high FiO_2_ was cost-effective at preventing SSIs. In Nigeria, the average cost for high FiO_2_ was $216 compared with $222 for low FiO_2_ leading to a difference in cost of −$6 (95% CI: −$13 to −$1). In India, the average cost for high FiO_2_ was $184 compared with $195 for low FiO_2_, leading to a difference in cost of −$11 (95% CI: −$15 to −$6). In South Africa, the average cost for high FiO_2_ was $1164 compared with $1257 for low FiO_2_, leading to a difference in cost of −$93 (95% CI −$132 to −$65). The higher cost difference in South Africa was driven by the high bed day and re-intervention costs compared with the other two countries. High FiO_2_ had smaller percentage of SSIs, 7.33% compared with 8.38% for low FiO_2_, leading to −1.05 (95% CI: −1.14 to − 0.90) percentage point reduction in SSIs (Table 3).

**Table 3:** Base-case cost-effectiveness results. All costs are in 2020 for US dollars. CI, confidence interval; FiO_2_, perioperative fraction of inspired oxygen; LMICs, low-and middle-income countries; N/A, not applicable. ∗Effectiveness measure for all countries.

	High FiO_2_	Low FiO_2_	Difference in costs (CI)	Difference in effects (95% CI)
Nigeria (cost)	$216	$222	−$6 (−$13 to −$1)	N/A
India (cost)	$184	$195	−$11 (−$15 to −$6)	N/A
South Africa (cost)	$1164	$1257	−$93 (−$132 to −$65)	N/A
SSIs, % (all∗)	7.33	8.38	N/A	−1.05 (−1.14 to −0.90)

### Deterministic sensitivity analysis

The results were sensitive to changes in model probabilities that were made in favour of low FiO_2_ (Supplementary Appendix S5).

### Probabilistic sensitivity analysis

**Fig 2:**
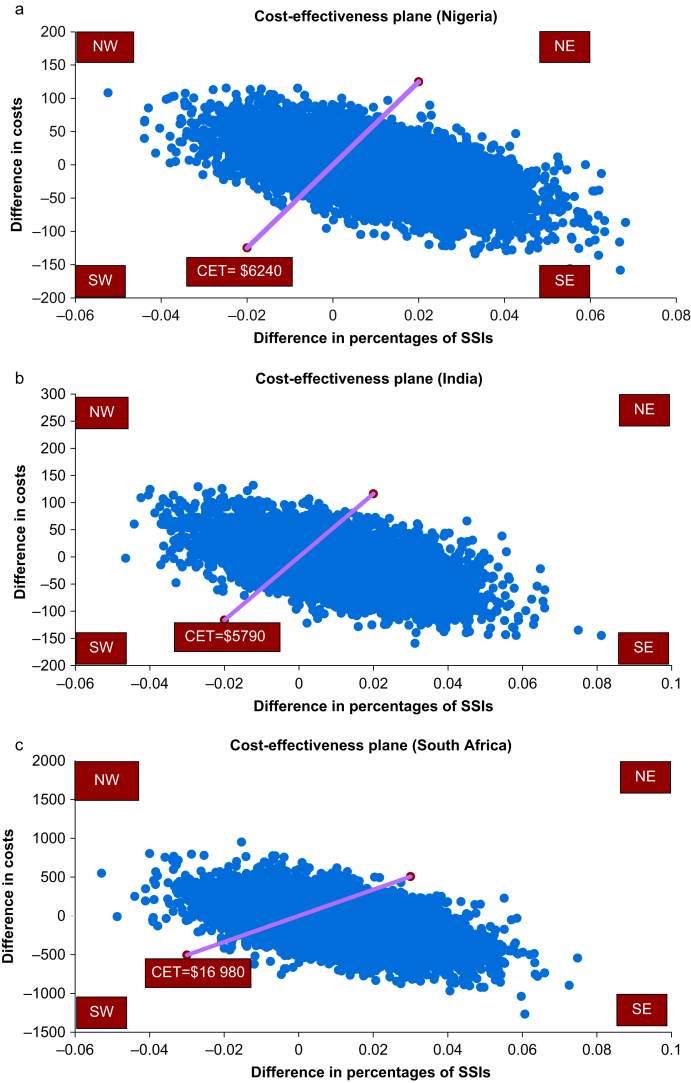
Cost-effectiveness planes. Four quadrants: NE stands for North-East (more effective and more costly), NW stands for North-West (less effective and more costly), SW stands for South-West (less effective and cheaper), and SE stands for South-East (more effective and cheaper). (a) Cost-effectiveness plane for Nigeria: 51% of the scatterplots were in the SE quadrant implying that more than half of the scatterplots indicated that the intervention is likely cost-effective. (b) Cost-effectiveness plane for India: 53% of the scatterplots were in the SE implying that more than half of the scatterplots indicated that the intervention is likely cost-effective. (c) Cost-effectiveness plane for South Africa: 56% of the scatterplots were in the SE implying that more than half of the scatterplots indicated that the intervention is likely cost-effective. CET, cost-effectiveness threshold; SSI, surgical site infection.

The cost-effectiveness plane plots difference in costs against difference in percentage of SSIs (Fig 2a–c). In all the three countries, most points are in the South-East (better outcomes and cheaper) and North-East (better outcomes but costly) quadrants suggesting that the intervention is more effective. However, there is uncertainty whether this effectiveness is associated with high costs (North-East quadrant) or low costs (South-East quadrant). At three times GDP per capita in all three countries, at least 50% of the points were below the threshold line indicating that high FiO_2_ was likely to be cost-effective.

**Fig 3:**
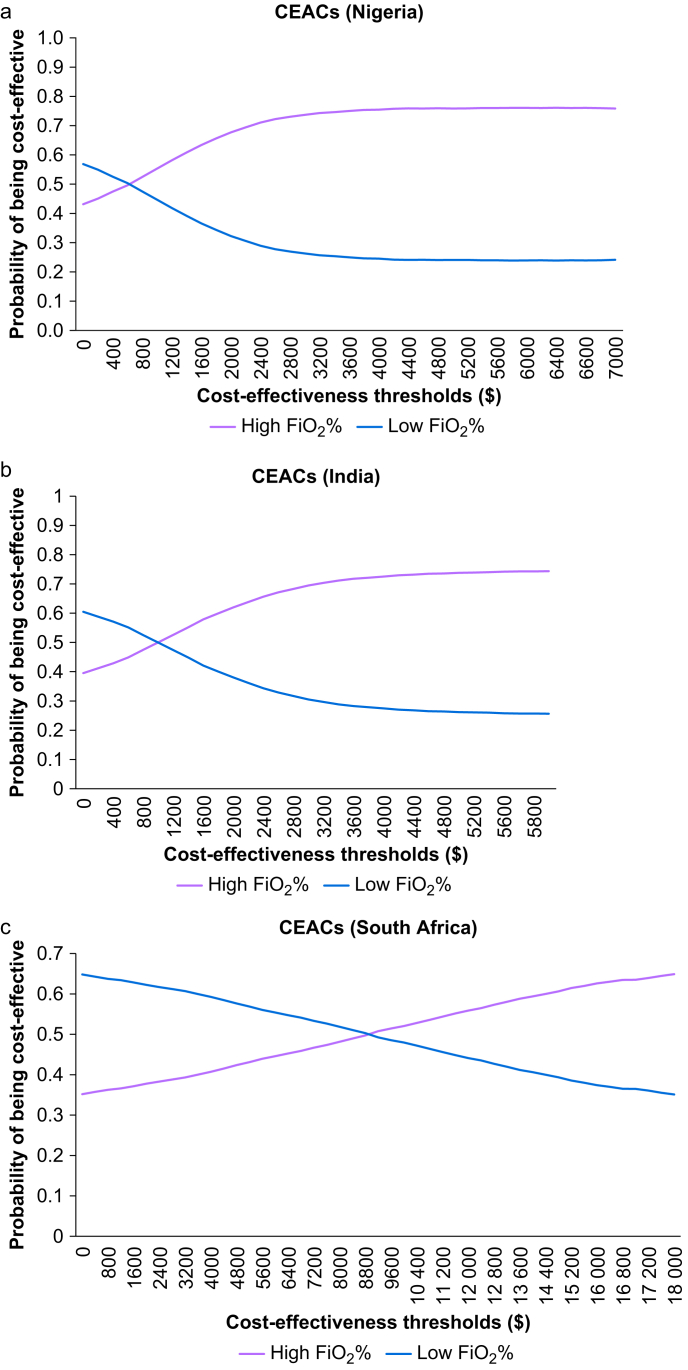
Cost-effectiveness acceptability curves for (a) Nigeria, (b) India, and (c) South Africa. CEACs, cost-effectiveness acceptability curves; FiO_2_, perioperative fraction of inspired oxygen.

The CEACs show the probability of high and low FiO_2_ being cost-effective at various CETs. At three times GDP per capita, high FiO_2_ had 0.76, 0.74, and 0.64 probability of being cost-effective compared with 0.24, 0.26, and 0.36 probability of low FiO_2_ being cost-effective in Nigeria, India, and South Africa, respectively, indicating that the intervention was likely to be cost-effective (Fig 3a–c).

## Discussion

Our analyses suggest that high FiO_2_ (compared with low FiO_2_) would be a cost-effective treatment at preventing SSIs in LMICs but further data from large clinical trials are required to confirm the clinical effectiveness of this approach. Our findings are based on current estimates of high FiO_2_ at preventing SSIs, MID, and unbundled data sourced from an ongoing international pilot trial in LMICs aimed at resolving the uncertainty on the effectiveness of high FiO_2_. The results appear to be driven by a reduction in postoperative complications that led to lower re-intervention rates (which had high costs in all three countries). The implication is that the slight increase in costs associated with high FiO_2_ would be outweighed by a reduction in hospital costs associated with re-intervention. Thus, if confirmed to be effective at reducing SSIs, high FiO_2_ is likely to benefit patients undergoing abdominal surgery in the three countries included in this evaluation and should be considered for implementation in surgical practice. However, this study does not assess the effectiveness of the intervention and it is possible that high FiO_2_ does not have clinical benefits with respect to SSI. Oxygen therapy is crucial to the management of many conditions including during and after surgery, however, oxygen supply systems are not robust in many LMICs.^25^ SSI treatment is expensive in both HICs and LMICs^26^ and there is a need to find effective and cost-effective interventions for preventing SSIs in order to utilise scarce healthcare resources efficiently.

Evidence from HICs show that other interventions for SSI prevention are cost-effective, which aligns with the current findings. In the UK, a single-centre study of patients undergoing elective colorectal resection established that oral antibiotic (neomycin and metronidazole) bowel preparation (OABP) plus mechanical bowel preparation (MBP) was more effective and cheaper at preventing SSIs compared with MBP alone. The OABP plus MBP arm had fewer SSIs compared with the MBP arm (16% *vs* 1.5% SSIs) and was cheaper (£94.84 *vs* £336.54 per patient).^27^ In the USA, a barrier wound protection device and a bundle of interventions to prevent SSIs were cost-effective at preventing SSIs among colorectal surgery patients when compared with standard practice. A single-ring wound protection device and a double-ring wound protection device were associated with $0.003 and $0.02 net monetary benefits (NMB) whereas the bundle was associated with $0.01 NMB. The interventions were considered cost-effective because they had a positive NMB.^28^

To our knowledge, this is the first study to evaluate the cost-effectiveness of high FiO_2_ for SSI prevention in any surgical patient group. There were limitations relating to availability of quality data. Firstly, this analysis did not consider other outcomes or adverse effects of high *vs* low FiO_2_. Secondly, South Africa costs are likely to be representative of the public facilities but might not reflect the private sector facilities, as they were sourced from public provincial and national reference fees. Thirdly, costs in Nigeria and India might not be representative because localised costs were sourced from few hospitals in the study which might not reflect country averages as the centralised costs used in studies in HICs. Fourthly, there were no thresholds for assessing outcomes presented as incidence, as such GDP-based thresholds were used. In theory, the GDP-based CETs should be used when the outcome measure is the disability adjusted life year. However, these thresholds have been used in published studies across a range of outcomes including natural units because of the lack of alternative thresholds.^24^^,^^29^^,^^30^ Fifthly, because FiO_2_ is scarce, hospitals especially in low resource settings may not be able to administer high FiO_2_ if clinical trials establish that high FiO_2_ is effective. Finally, this study did not assess the cost-effectiveness of other interventions in different areas that use oxygen, so we do not know whether we are making the best use of the resource constraint (oxygen). However, given that the cost of providing oxygen may not equal its opportunity cost, we have varied the high FiO_2_ cost in a threshold analysis to assess the impact on results. The sensitivity analysis indicates that if high FiO_2_ was 7% more expensive, there would be no cost savings from the intervention.

Perioperative interventions such as perioperative high FiO_2_, antibiotic prophylaxis, antiseptic prophylaxis, perioperative glycaemic control, and maintaining normal body temperature during the perioperative period have been recommended for SSI prevention.^4^^,^^7^^,^^31^ However, there are uncertainties and ongoing debates on the effectiveness and safety of perioperative high FiO_2_. The cost-effectiveness of interventions differs depending on setting and the recommended SSI prevention interventions can be cost-effective in some, but not all countries.^11^ This study has shown the potential cost-effectiveness of high FiO_2_ at preventing SSIs among abdominal surgery patients in Nigeria, India, and South Africa. Therefore, high FiO_2_ should be considered as a future alternative for preventing SSIs in Nigeria, India, and South Africa if clinical effectiveness of the intervention is shown in the PENGUIN Trial. Further, there is a need to prioritise improving oxygen supply systems in LMICs by adopting more effective and less costly anaesthetic interventions.

The results suggest that high FiO_2_ is cheaper and more effective at reducing SSIs among abdominal surgery patients in Nigeria, India, and South Africa. These are LMICs with high SSI rates of between 20% and 31%.^32^, ^33^, ^34^ However, because of limitations, assumptions were made regarding effectiveness of high FiO_2_ which highlight the need for clinical trials to assess the effectiveness and safety of high FiO_2_ at preventing SSIs. If the ongoing PENGUIN clinical trial establishes that high FiO_2_ is effective and safe, it will be worthwhile for high FiO_2_ to be used routinely in surgical practice in the three countries.

## Writing committee members' contributions

Chairing Writing Committee, writing first draft and editing the manuscript: MK.

Model construction: MK, JG.

Data analysis: MK, MM, RO, TER.

Study conceptualisation: MM, RO, TER, BMB, DGM, RP, YMA, OO, SYY, AOA.

Data analysis supervision: MM, RO, TER.

Manuscript preparation: all authors.

Data preparation: BMB.

GlobalSurg-2 observational cohort study data preparation: JG.

Read the manuscript and approved it for submission: all authors.

## Collaborators

### South Africa:

Bruce M. Biccard, Denton Smith and Shrikant Peters, Adam Boutall, Graeme Wilson, Ettienne Coetzee, Margot Flint, Simphiwe Gumede, Shreya Rayamajhi, Sharon Bannister, Nonkululo Daniel (Groote Schuur Hospital).

Maria Fourtounas, Rachel Moore, Nnosa Sentholang (Chris Hani Baragwaneth Hospital).

### Nigeria:

Osaheni Osayomwanbo (University of Benin Teaching Hospital), Aghadi Ifeanyi kene (Barau Dikko University Teaching Hospital), Saidu Yusuf Yakubu, Amos Chukwu and Musliu Tolani (Ahmadu Bello University Teaching Hospital Zaria), Yakubu Momohsani Adinoyi (Federal Medical Centre Birnin Kebbi), Abdulrahman Aliyu (Uduth Sokoto Hospital), Dalhat Salahu (Aminu Kano Teaching Hospital), Isa kabir (Federal Medical Centre Gusau), Ibrahim Salisu (Federal Medical Centre Katsina), Tinuola Adigun (University College Hospital Ibadan), Anthony Adenekan (Obafemi Awolowo University Teaching Hospital), Emmanuel Williams (NIHR Global Health Research Unit on Global Surgery, Nigeria).

### India:

Pradeep Kumar Bhatia, Ramkaran Chaudhary, Nikhil Kothari, Sanjeev Misra, Puneet Pareek, Dharma Ram Poonia, Kirti Kumar Rathod, Mahaveer Singh Rodha, Naveen Sharma, Nivedita Sharma, Subhash Chandra Soni, Vaibhav Kumar Varshney, Jeewan Ram Vishnoi (All India Institute of Medical Science, Jodhpur).

Satya Shree Balija, Anuj Goyal, Farhanul Hudda, Manoji Joshva, Rajkumar Kottayasamy Seenivasagam, Shafiq Shajahan, Sameer Sharma, Sunil Kumar Singh, Praveen Talwar, Debendra Kumar Tripathi (All India Institute of Medical Science, Rishikesh).

Bhatt, Swati Daniel, Jyoti Dhiman, Christina George, Dhruva N Ghosh, Sunita Goyal, Priyanka Hans, Parvez D Haque, Deepak Jain, Harsharan Kaur, Karan Kumar, Amit Mahajan, Vishal Michael, Reuben Rajappa, Arti Rajkumar, Atul Suroy, Ravinder Singh Thind, Sreejith K Veetil (Christian Medical College & Hospital, Ludhiana) Alisha Manisha Aggarwal, Parth Dhamija, Gurleen Kaur Garry, Himani Gupta, Ruchi Jakhar, Ashwani Kumar, Kshitij Kumar, Parmod Kumar, Gurtaj Singh (Government Medical College and Rajindra Hospital Patiala).

Sona Chowdhury, Neha Desai, Jyotsna Goswami, Sonia Mathai, Viplab Patro (Tata Medical College, Kolkata).

## Funding

The 10.13039/501100000272National Institute for Health Research (10.13039/501100000272NIHR) (10.13039/501100000272NIHR 16.136.79) using 10.13039/100007397UK aid from the 10.13039/100013986UK Government to support global health research. The views expressed in this publication are those of the author(s) and not necessarily those of the NIHR or the UK Department of Health and Social Care.

## Declarations of interest

RP holds research grants, honoraria, or both from 10.13039/100006520Edwards Lifesciences, Intersurgical, and 10.13039/100004330GlaxoSmithKline, and is a member of the editorial board of the *British Journal of Anaesthesia*. The rest of the writing committee members declare that they have no conflicts of interest.

## References

[1] Mueller T.C., Loos M., Haller B. (2015). Intra-operative wound irrigation to reduce surgical site infections after abdominal surgery: a systematic review and meta-analysis. Langenbecks Arch Surg.

[2] Bhangu A., Ademuyiwa A.O., Aguilera M.L. (2018). Surgical site infection after gastrointestinal surgery in high-income, middle-income, and low-income countries: a prospective, international, multicentre cohort study. Lancet Infect Dis.

[3] Dégbey C., Kpozehouen A., Coulibaly D. (2021). Prevalence and factors associated with surgical site infections in the university clinics of traumatology and urology of the national university hospital centre hubert koutoukou maga in cotonou. Front Public Health.

[4] Allegranzi B., Zayed B., Bischoff P. (2016). New WHO recommendations on intraoperative and postoperative measures for surgical site infection prevention: an evidence-based global perspective. Lancet Infect Dis.

[5] Usher A.D. (2021). Medical oxygen crisis: a belated COVID-19 response. Lancet.

[6] Myles P.S., Carlisle J.B., Scarr B. (2019). Evidence for compromised data integrity in studies of liberal peri-operative inspired oxygen. Anaesthesia.

[7] World Health Organization (2018).

[8] Mattishent K., Thavarajah M., Sinha A. (2019). Safety of 80% vs 30–35% fraction of inspired oxygen in patients undergoing surgery: a systematic review and meta-analysis. Br J Anaesth.

[9] NIHR Health Research Unit on Global Surgery (2020). https://clinicaltrials.gov/ct2/show/NCT04256798.

[10] Briggs A.H., Karl Claxton, Sculpher Mark J. (2011).

[11] Drummond M.F., Sculpher M.J., Claxton K., Stoddart G.L., Torrance G.W. (2015).

[12] Barton P., Bryan S., Robinson S. (2004). Modelling in the economic evaluation of health care: selecting the appropriate approach. J Health Serv Res Policy.

[13] Centers for Disease Control and Prevention Surgical site infection event (SSI). https://www.cdc.gov/nhsn/pdfs/pscmanual/9pscssicurrent.pdf2023.

[14] Harris P.A., Taylor R., Thielke R., Payne J., Gonzalez N., Conde J.G. (2009). Research electronic data capture (REDCap) – a metadata-driven methodology and workflow process for providing translational research informatics support. J Biomed Inform.

[15] Biccard B.M., Madiba T.E., Kluyts H.L. (2018). Perioperative patient outcomes in the African Surgical Outcomes Study: a 7-day prospective observational cohort study. Lancet.

[16] GlobalSurg C. (2018). Surgical site infection after gastrointestinal surgery in high-income, middle-income, and low-income countries: a prospective, international, multicentre cohort study. Lancet Infect Dis.

[17] Republic of South Africa Department of Health Uniform patient fee schedule CODE BOOK 2020. https://www.health.gov.za/uniform-patient-fee-schedule/.

[18] Schulman K., Burke J., Drummond M. (1998). Resource costing for multinational neurologic clinical trials: methods and results. Health Econ.

[19] NIHR Global Research Health Unit on Global Surgery (2021). Reducing surgical site infections in low-income and middle-income countries (FALCON): a pragmatic, multicentre, stratified, randomised controlled trial. Lancet.

[20] World Bank (2020). https://data.worldbank.org/indicator/PA.NUS.PPP.

[21] (2020). Inflation calculator.

[22] Glick H.A., Doshi J.A., Sonnad S.S., Polsky D. (2015).

[23] Saramago P., Manca A., Sutton A.J. (2012). Deriving input parameters for cost-effectiveness modeling: taxonomy of data types and approaches to their statistical synthesis. Value Health.

[24] Carvalho A.C., Leal F., Sasse A.D. (2017). Cost-effectiveness of cetuximab and panitumumab for chemotherapy-refractory metastatic colorectal cancer. PLoS One.

[25] Duke T., Wandi F., Jonathan M. (2008). Improved oxygen systems for childhood pneumonia: a multihospital effectiveness study in Papua New Guinea. Lancet.

[26] Monahan M., Jowett S., Pinkney T. (2020). Surgical site infection and costs in low- and middle-income countries: a systematic review of the economic burden. PloS One.

[27] Vadhwana B., Pouzi A., Surjus Kaneta G. (2020). Preoperative oral antibiotic bowel preparation in elective resectional colorectal surgery reduces rates of surgical site infections: a single-centre experience with a cost-effectiveness analysis. Ann R Coll Surg Engl.

[28] Chomsky-Higgins K., Kahn J.G. (2019). Interventions and innovation to prevent surgical site infection in colorectal surgery: a cost-effectiveness analysis. J Surg Res.

[29] World Health Organisation (2003).

[30] Shim E., Hampson K., Cleaveland S., Galvani A.P. (2009). Evaluating the cost-effectiveness of rabies post-exposure prophylaxis: a case study in Tanzania. Vaccine.

[31] Berríos-Torres S.I., Umscheid C.A., Bratzler D.W. (2017). Centers for Disease control and prevention guideline for the prevention of surgical site infection. JAMA Surg.

[32] Swart O., Esterhuizen T., Voss M. (2021). The role of treatment delays in surgical site infection after appendicectomy in a South African rural regional hospital. S Afr Med J.

[33] Nwankwo E., Ibeh I., Enabulele O. (2012). Incidence and risk factors of surgical site infection in a tertiary health institution in Kano, Northwestern Nigeria. Int J Infect Control.

[34] Kamat U.S., Fereirra A.M., Kulkarni M.S., Motghare D.D. (2008). A prospective study of surgical site infections in a teaching hospital in Goa. Indian J Surg.

