# Erratum to “Exploring the cost-effectiveness of high versus low perioperative fraction of inspired oxygen in the prevention of surgical site infections among abdominal surgery patients in three low- and middle-income countries” [*BJA Open* 7 (2023) 100207]

**DOI:** 10.1016/j.bjao.2023.100251

**Published:** 2024-02-28

**Authors:** Mwayi Kachapila, Mark Monahan, Adesoji O. Ademuyiwa, Yakubu Momohsani Adinoyi, Bruce M. Biccard, Christina George, Dhruva N. Ghosh, James Glasbey, Dion G. Morton, Osaheni Osayomwanbo, Rupert Pearse, Tracy E. Roberts, Atul Suroy, Saidu Yusuf Yakubu, Raymond Oppong, NIHR Global Health Research Unit on Global Surgery Collaborators

**Affiliations:** 1 NIHR Global Health Research Unit on Global Surgery, University of Birmingham, Birmingham, UK; 2 Health Economics Unit, University of Birmingham, Birmingham, UK; 3 Paediatric Surgery Unit, Department of Surgery, Faculty of Clinical Sciences, College of Medicine, University of Lagos, Lagos, Nigeria; 4 Federal Medical Centre, Birnin Kebbi, Nigeria; 5 Department of Anaesthesia and Perioperative Medicine, Groote Schuur Hospital and University of Cape Town, Cape Town, South Africa; 6 India Hub NIHR Global Health Research Unit on Global Surgery, Ludhiana, India; 7 Department of Anaesthesia and Surgery, Christian Medical College, Ludhiana, India; 8 Birmingham Surgical Trials Consortium, University of Birmingham, Birmingham, UK; 9 University of Benin Teaching Hospital, Benin City, Edo, Nigeria; 10 Faculty of Medicine and Dentistry, Queen Mary University of London, London, UK; 11 Ahmadu Bello University Teaching Hospital Zaria, Zaria, Nigeria

The publisher regrets that the author details for this article were incomplete. The full list of authors and affiliations appears above. The acknowledgements section at the end of the article should be as follows:

