# Erratum to ‘Exploring the cost-effectiveness of high versus low perioperative fraction of inspired oxygen in the prevention of surgical site infections among abdominal surgery patients in three low- and middle-income countries’ [*BJA Open* 7 (2023) 100207]

**DOI:** 10.1016/j.bjao.2024.100267

**Published:** 2024-03-06

**Authors:** Mwayi Kachapila, Mark Monahan, Adesoji O. Ademuyiwa, Yakubu Momohsani Adinoyi, Bruce M. Biccard, Christina George, Dhruva N. Ghosh, James Glasbey, Dion G. Morton, Osaheni Osayomwanbo, Rupert Pearse, Tracy E. Roberts, Atul Suroy, Saidu Yusuf Yakubu, Raymond Oppong, NIHR Global Health Research Unit on Global Surgery Collaborators, Bruce M. Biccard, Denton Smith, Shrikant Peters, Adam Boutall, Graeme Wilson, Ettienne Coetzee, Margot Flint, Simphiwe Gumede, Shreya Rayamajhi, Sharon Bannister, Nonkululo Daniel, Maria Fourtouna, Rachel Moore, Nnosa Sentholang, Osaheni Osayomwanbo, Aghadi Ifeanyi kene, Saidu Yusuf Yakubu, Amos Chukwu, Musliu Tolani, Yakubu Momohsani Adinoyi, Abdulrahman Aliyu, Dalhat Salahu, Isa kabir, Ibrahim Salisu, Tinuola Adigun, Anthony Adenekan, Emmanuel Williams, Pradeep Kumar Bhatia, Ramkaran Chaudhary, Nikhil Kothari, Sanjeev Misra, Puneet Pareek, Dharma Ram Poonia, Kirti Kumar Rathod, Mahaveer Singh Rodha, Naveen Sharma, Nivedita Sharma, Subhash Chandra Soni, Vaibhav Kumar Varshney, Jeewan Ram Vishnoi, Satya Shree Balija, Anuj Goyal, Farhanul Hudda, Manoji Joshva, Rajkumar Kottayasamy Seenivasagam, Shafiq Shajahan, Sameer Sharma, Sunil Kumar Singh, Praveen Talwar, Debendra Kumar Tripathi, Bhatt, Swati Daniel, Jyoti Dhiman, Christina George, Dhruva N. Ghosh, Sunita Goyal, Priyanka Hans, Parvez D. Haque, Deepak Jain, Harsharan Kaur, Karan Kumar, Amit Mahajan, Vishal Michael, Reuben Rajappa, Arti Rajkumar, Atul Suroy, Ravinder Singh Thind, Sreejith K. Veetil, Alisha Manisha Aggarwal, Parth Dhamija, Gurleen Kaur Garry, Himani Gupta, Ruchi Jakhar, Ashwani Kumar, Kshitij Kumar, Parmod Kumar, Gurtaj Singh, Sona Chowdhury, Neha Desai, Jyotsna Goswami, Sonia Mathai, Viplab Patro

**Affiliations:** 12 Groote Schuur Hospital, South Africa; 13 Chris Hani Baragwaneth Hospital, South Africa; 14 University of Benin Teaching Hospital, Nigeria; 15 Barau Dikko University Teaching Hospital, Nigeria; 16 Ahmadu Bello University Teaching Hospital Zaria, Nigeria; 17 Federal Medical Centre Birnin Kebbi, Nigeria; 18 Uduth Sokoto Hospital, Nigeria; 19 Aminu Kano Teaching Hospital, Nigeria; 20 Federal Medical Centre Gusau, Nigeria; 21 Federal Medical Centre Katsina, Nigeria; 22 University College Hospital Ibadan, Nigeria; 23 Obafemi Awolowo University Teaching Hospital, Nigeria; 24 NIHR Global Health Research Unit on Global Surgery, Nigeria; 25 India Institute of Medical Science, Jodhpur, India; 26 India Institute of Medical Science, Rishikesh, India; 27 Christian Medical College & Hospital, Ludhiana, India; 28 Government Medical College and Rajindra Hospital Patiala, India; 29 Tata Medical College, Kolkata, India; 1 NIHR Global Health Research Unit on Global Surgery, University of Birmingham, Birmingham, UK; 2 Health Economics Unit, University of Birmingham, Birmingham, UK; 3 Paediatric Surgery Unit, Department of Surgery, Faculty of Clinical Sciences, College of Medicine, University of Lagos, Lagos, Nigeria; 4 Federal Medical Centre, Birnin Kebbi, Nigeria; 5 Department of Anaesthesia and Perioperative Medicine, Groote Schuur Hospital and University of Cape Town, Cape Town, South Africa; 6 India Hub NIHR Global Health Research Unit on Global Surgery, Ludhiana, India; 7 Department of Anaesthesia and Surgery, Christian Medical College, Ludhiana, India; 8 Birmingham Surgical Trials Consortium University of Birmingham, Birmingham, UK; 9 University of Benin Teaching Hospital, Benin City, Edo, Nigeria; 10 Faculty of Medicine and Dentistry, Queen Mary University of London, London, UK; 11 Ahmadu Bello University Teaching Hospital Zaria, Zaria, Nigeria

The publisher regrets that errors were present in the author and collaborator details. The full list of authors and affiliations appears above, and the amended collaborator list and acknowledgements are below. Please note that Appendix S1 accompanying the original article has also been updated to include all members of the GlobalSurg Collaborative.

^**†**^ **NIHR Global Health Research Unit on Global Surgery Collaborators**

**South Africa**

Bruce M. Biccard, Denton Smith, Shrikant Peters, Adam Boutall, Graeme Wilson, Ettienne Coetzee, Margot Flint, Simphiwe Gumede, Shreya Rayamajhi, Sharon Bannister, Nonkululo Daniel (Groote Schuur Hospital).

Maria Fourtounas, Rachel Moore, Nnosa Sentholang (Chris Hani Baragwaneth Hospital).

**Nigeria**

Osaheni Osayomwanbo (University of Benin Teaching Hospital).

Aghadi Ifeanyi Kene (Barau Dikko University Teaching Hospital).

Saidu Yusuf Yakubu, Amos Chukwu and Musliu Tolani (Ahmadu Bello University Teaching Hospital Zaria).

Yakubu Momohsani Adinoyi (Federal Medical Centre Birnin Kebbi).

Abdulrahman Aliyu (Uduth Sokoto Hospital).

Dalhat Salahu (Aminu Kano Teaching Hospital).

Isa Kabir (Federal Medical Centre Gusau).

Ibrahim Salisu (Federal Medical Centre Katsina).

Tinuola Adigun (University College Hospital Ibadan).

Anthony Adenekan (Obafemi Awolowo University Teaching Hospital).

Emmanuel Williams (NIHR Global Health Research Unit on Global Surgery, Nigeria).

**India**

Pradeep Kumar Bhatia, Ramkaran Chaudhary, Nikhil Kothari, Sanjeev Misra, Puneet Pareek, Dharma Ram Poonia, Kirti Kumar Rathod, Mahaveer Singh Rodha, Naveen Sharma, Nivedita Sharma, Subhash Chandra Soni, Vaibhav Kumar Varshney, Jeewan Ram Vishnoi (India Institute of Medical Science, Jodhpur).

Satya Shree Balija, Anuj Goyal, Farhanul Hudda, Manoji Joshva, Rajkumar Kottayasamy Seenivasagam, Shafiq Shajahan, Sameer Sharma, Sunil Kumar Singh, Praveen Talwar, Debendra Kumar Tripathi (India Institute of Medical Science, Rishikesh).

Bhatt, Swati Daniel, Jyoti Dhiman, Christina George, Dhruva N Ghosh, Sunita Goyal, Priyanka Hans, Parvez D Haque, Deepak Jain, Harsharan Kaur, Karan Kumar, Amit Mahajan, Vishal Michael, Reuben Rajappa, Arti Rajkumar, Atul Suroy, Ravinder Singh Thind, Sreejith K Veetil (Christian Medical College & Hospital, Ludhiana). Alisha Manisha Aggarwal, Parth Dhamija, Gurleen Kaur Garry, Himani Gupta, Ruchi Jakhar, Ashwani Kumar, Kshitij Kumar, Parmod Kumar, Gurtaj Singh (Government Medical College and Rajindra Hospital Patiala).

Sona Chowdhury, Neha Desai, Jyotsna Goswami, Sonia Mathai, Viplab Patro (Tata Medical College, Kolkata**).**

**Acknowledgements**

We acknowledge all members of the GlobalSurg Collaborative (listed in Appendix S1) without whom this work would not have been possible.

The publisher would like to apologise for any inconvenience caused.

