# Supplementary material for: Exploring the cost-effectiveness of high versus low perioperative fraction of inspired oxygen in the prevention of surgical site infections among abdominal surgery patients in three low- and middle-income countries
Source: BJA Open. 2023 Jul 15;7:100207. doi: 10.1016/j.bjao.2023.100207 (PMC10457493; doi:10.1016/j.bjao.2023.100207)
Supplement: Multimedia component 1 [file mmc1.docx]

**Appendix S1:**

**Membership of the GlobalSurg Collaborative**

*GlobalSurg Collaborative writing group*: Aneel Bhangu, Adesoji O Ademuyiwa, Maria Lorena Aguilera, Philip Alexander, Sara W Al-Saqqa, Giuliano Borda-Luque, Ainhoa Costas-Chavarri, Thomas M Drake, Faustin Ntirenganya, J Edward Fitzgerald, Stuart J Fergusson, James Glasbey, J.C Allen Ingabire, Lawani Ismaïl, Hosni Khairy Salem, Anyomih Theophilus Teddy Kojo, Marie Carmela Lapitan, Richard Lilford, Andre L Mihaljevic, Dion Morton, Alphonse Zeta Mutabazi, Dmitri Nepogodiev, Adewale O Adisa, Riinu Ots, Francesco Pata, Thomas Pinkney, Tomas Poškus, Ahmad Uzair Qureshi, Antonio Ramos-De la Medina, Sarah Rayne, Catherine A Shaw, Sebastian Shu, Richard Spence, Neil Smart, Stephen Tabiri, Ewen M Harrison

*GlobalSurg Collaborative patient representatives*: Azmina Verjee, Emmy Runigamugabo

*Protocol development*: Chetan Khatri, Midhun Mohan, Thomas M Drake, James Glasbey, Dmitri Nepogodiev, Catherine A Shaw, Zahra Jaffry, Stuart J Fergusson, Francesco Pata, Adesoji O Ademuyiwa, Afnan Altamini, Hosni Khairy Salem, Andrew Kirby, Kjetil Søreide, Gustavo Recinos, Richard Spence, Sarah Rayne, Stephen Tabiri, Jen Cornick, Thomas Pinkney, Richard Lilford, J Edward Fitzgerald, Ewen M Harrison, Aneel Bhangu

*GlobalSurg Collaborative national leads*:

**Argentina,** Maria Marta Modolo; **Australia**, Dushyant Iyer, Sebastian King, Tom Arthur; **Bangladesh,** Sayeda Nazmum Nahar; **Barbados,** Ade Waterman; **Benin,** Lawani Ismaïl; **Botswana,** Michael Walsh; **Canada,** Arnav Agarwal, Augusto Zani, Mohammed Firdouse, Tyler Rouse; **China,** Qinyang Liu; **Colombia,** Juan Camilo Correa; **Egypt,** Hosni Khairy Salem; **Estonia,** Peep Talving; **Ethiopia,** Mengistu Worku; **France**, Alexis Arnaud; **Ghana,** Stephen Tabiri; **Greece,** Vassilis Kalles; **Guatemala**, Maria Lorena Aguilera, Gustavo Recinos; **India**, Basant Kumar, Sunil Kumar; **Indonesia**, Radhian Amandito; **Ireland**, Roy Quek; **Italy**, Francesco Pata, Luca Ansaloni; **Jordan**, Ahmed Altibi; **Lithuania**, Donatas Venskutonis, Justas Zilinskas, Tomas Poskus; **Madagascar**, John Whitaker; **Malawi**, Vanessa Msosa; **Malaysia**, Yong Yong Tew; **Malta**, Alexia Farrugia, Elaine Borg; **Mexico**, Antonio Ramos-De la Medina; **Morocco**, Zineb Bentounsi; **Nigeria**, Adesoji O Ademuyiwa; **Norway**, Kjetil Søreide; **Pakistan,** Tanzeela Gala; **Palestinian Territory,** Ibrahim Al-Slaibi, Haya Tahboub, Osaid H. Alser; **Peru,** Diego Romani, Sebestian Shu; **Poland,** Piotr Major; **Romania**, Aurel Mironescu, Matei Bratu, Amar Kourdouli; **Saint Kitts and Nevis,** Aliyu Ndajiwo; **Saudi Arabia,** Abdulaziz Altwijri, Mohammed Ubaid Alsaggaf, Ahmad Gudal, Al Faifi Jubran; **Sierra Leone,** Sam Seisay; **Singapore,** Bettina Lieske; **South Africa,** Sarah Rayne, Richard Spence; **Spain,** Irene Ortega; **Sri Lanka,** Jenifa Jeyakumar, Kithsiri J. Senanayake; **Sudan,** Omar Abdulbagi; **Sweden,** Yucel Cengiz; **Switzerland,** Dmitri Raptis; **Turkey,** Yuksel Altinel; **United Kingdom,** Chia Kong, Ella Teasdale, Gareth Irwin, Michael Stoddart, Rakan Kabariti, Sukrit Suresh; **United States,** Katherine Gash, Ragavan Narayanan; **Zambia,** Mayaba Maimbo

*GlobalSurg Collaborative Local collaborators:*

**Albania:** Besmir Grizhja, Shpetim Ymeri, Gezim Galiqi (Spitali Rajonal Shkoder).

**Argentina:** Roberto Klappenbach, Diego Antezana, Alvaro Enrique Mendoza Beleño, Cecilia Costa, Belen Sanchez, Susan Aviles (Hospital Zonal General De Agudos Simplemente Evita); Maria Marta Modolo, Claudio Gabriel Fermani, Rubén Balmaceda, Santiago Villalobos, Juan Manuel Carmona (Hospital Luis C. Lagomaggiore, Mendoza).

**Australia**: Daniel Hamill, Peter Deutschmann, Simone Sandler, Daniel Cox (Alice Springs Hospital); Ram Nataraja, Claire Sharpin, Damir Ljuhar (Monash Medical Center); Demi Gray, Morgan Haines (Port Macquarie Base Hospital); Dush Iyer, Nithya Niranjan, Scott D'Amours (Liverpool Hospital); Morvarid Ashtari, Helena Franco, (Gold Coast University Hospital).

**Bangladesh**: Ashrarur Rahman Mitul, Sabbir Karim, (Dhaka Shishu (Children) Hospital); Nowrin F. Aman, Mahnuma Mahfuz Estee (Holy Family Red Crescent Medical College & Hospital); Umme Salma, Joyeta Razzaque, Tasnia Hamid Kanta (Dhaka Medical College And Hospital); Sayeeda Aktar Tori, Shadid Alamin, Swapnil Roy, Shadid Al Amin, Rezaul Karim (Armed Forces Medical College); Muhtarima Haque, Amreen Faruq, Farhana Iftekhar (Birdem Bangladesh Institute Of Research And Rehabilitation In Diabetes Endocrine And Metabolic Disorder General Hospital);

**Barbados**: Margaret O'Shea, Greg Padmore, Ramesh Jonnalagadda (Queen Elizabeth Hospital).

**Belarus**: Andrey Litvin, Aliaksandr Filatau, Dzmitry Paulouski, Maryna Shubianok, Tatsiana Shachykava (Gomel Regional Clinical Hospital); Dzianis Khokha, Vladimir Khokha (City Hospital)

**Benin**: Fernande Djivoh, Lawani Ismaïl, Francis Dossou (Centre National Hospitalier Et Universitaire Hubert Koutoukou Maga); Djifid Morel Seto, Dansou Gaspard Gbessi, Bruno Noukpozounkou, Yacoubou Imorou Souaibou, (Centre National Hospitalier Et Universitaire Hubert Koutoukou Maga); Kpèmahouton René Keke, Fred Hodonou (Clinique Vignon); Ernest Yemalin Stephane Ahounou, Thierry Alihonou (Hopital El Fateh); Max Dénakpo, Germain Ahlonsou (Hospital Saint Luc).

**Botswana**: Alemayehu Ginbo Bedada (Princess Marina Hospital).

**Burundi**: Carlos Nsengiyumva, Sandrine Kwizera, Venerand Barendegere (Hopital Militaire De Kamenge).

**Cambodia**: Philip Choi, Simon Stock (World Mate Emergency Hospital)

**Canada**: Luai Jamal, Mohammed Firdouse, Augusto Zani, Georges Azzie, Sameer Kushwaha, Arnav Agarwal (The Hospital for Sick Children).

**China**: Tzu-Ling Chen, Chingwan Yip (Fudan University Affiliated Huashan Hospital).

**Colombia**: Irene Montes, Felipe Zapata, Sebastian Sierra (Clinica CES); Maria Isabel Villegas Lanau, Maria Clara Mendoza Arango, Ivan Mendoza Restrepo (Clinica Las Vegas), Sebastian Sierra, Ruben Santiago Restrepo Giraldo, Maria Clara Mendoza Arango (Hospital Universitario San Vicente Fundación).

**Croatia**: Edgar Domini, Robert Karlo, Jakov Mihanovic (Zadar General Hospital).

**Egypt**: Mohamed Youssef, Hossam Elfeki, Waleed Thabet, Aly Sanad, Gehad Tawfik, Ahmed Zaki, Noran Abdel-Hameed, Mohamed Mostafa, Muhammad Fathi Waleed Omar, Ahmed Ghanem, Emad Abdallah, Adel Denewar, Eman Emara, Eman Rashad, Ahmad Sakr, Rehab Elashry, Sameh Emile (Mansoura University Hospital); Toqa Khafagy, Sara Elhamouly, Arwa Elfarargy, Amna Mamdouh Mohamed, Ghada Saied Nagy, Abeer Esam, Eman Elwy, Aya Hammad, Salwa Khallaf, Eman Ibrahim, Ahmed Saidbadr, Ahmed Moustafa, Amany Eldosouky Mohammed, Mohammed Elgheriany, Eman Abdelmageed, Eman Abd Al Raouf, Esraa Samir Elbanby, Maha Elmasry, Mahitab Morsy Farahat, Eman Yahya Mansor, Eman Magdy Hegazy, Esraa Gamal, Heba Gamal, Hend Kandil, Doaa Maher Abdelrouf, Mohamed Moaty,(Menofiya University Hospital); Dina Gamal, Nada El-Sagheer, Mohamed Salah, Salma Magdy, Asmaa Salah, Ahmed Essam, Ahmed Ali, Mahmoud Badawy, Sara Ahmed, (Beni Suef University Hospital); Mazed Mohamed, Abdelrahman Assal, Mohamed Sleem, Mai Ebidy, Aly Abd Elrazek, Diaaaldin Zahran, Nourhan Adam, Mohamed Nazir, Adel B Hassanein, Ahmed Ismail, Amira Elsawy, Rana Mamdouh, Mohamed Mabrouk, Lopna Ahmed Mohamed Ahmed, Mohamed Hassab Alnaby, Eman Magdy, Manar Abd-Elmawla, Marwan Fahim, Bassant Mowafy, Moustafa Ibrahim Mahmoud, Meran Allam, Muhammad Alkelani, Noran Halim El Gendy, Mariam Saad Aboul-Naga, Reham Alaa El-Din, Alyaa Halim Elgendy, Mohamed Ismail, Mahmoud Shalaby, Aya Adel Elsharkawy, Mahmoud Elsayed Moghazy, Khaled Hesham Elbisomy, Hend Adel Gawad Shakshouk, Mohamed Fouad Hamed, Mai Mohamed Ebidy, Mostafa Abdelkader, Mohamed Karkeet (Alexandria Main UniversIty Hospital); Hayam Ahmed, Israa Adel, Mohammad Elsayed Omar, Mohamed Ibrahim, Omar Ghoneim, Omar Hesham, Shimaa Gamal, Karim Hilal, Omar Arafa, Sawsan Adel Awad, Menatalla Salem, Fawzia Abdellatif Elsherif, Nourhan Elsabbagh, Moustafa R. Aboelsoud, Ahmed Hossam Eldin Fouad Rida, Amr Hossameldin, Ethar Hany, Yomna Hosny Asar, Nourhan Anwar, Mohamed Gadelkarim, Samar Abdelhady, Eman Mohamed Morshedy, Reham Saad, Nourhan Soliman, Mahmoud Salama (Alexandria Medical Research Institute); Eslam Ezzat, Arwa Mohamed, Arwa Ibrahim, Alaa Fergany, Sara Mohammed, Aya Reda, Yomna Allam, Hanan Adel Saad, Afnan Abdelfatah, Aya Mohamed Fathy, Ahmed El-Sehily, Esraa Abdalmageed Kasem, Ahmed Tarek Abdelbaset Hassan, Ahmed Rabeih Mohammed, Abdalla Gamal Saad, Yasmin Elfouly, Nesma Elfouly, Arij Ibrahim, Amr Hassaan, Mohammed Mustafa Mohammed, Ghada Elhoseny, Mohamed Magdy, Esraa Abd Elkhalek, Yehia Zakaria, Tarek Ezzat, Ali Abo El Dahab , Mohamed Kelany, Sara Arafa, Osama Mokhtar Mohamed Hassan, Nermin Mohamed Badwi, Ahmad Saber Sleem, Hussien Ahmed, Kholoud Abdelbadeai, Mohamed Abozed Abdullah (Faculty Of Medicine, Zagazig University); Muhammad Amsyar Auni Lokman, Suraya Bahar, Anan Rady Abdelazeam, Abdelrahman Adelshone, Muhammad Bin Hasnan, Athirah Zulkifli, Siti Nur Alia Kamarulzamil, Abdelaziz Elhendawy, Aliang Latif, Ahmad Bin Adnan, Shahadatul Shaharuddin, Aminah Hanum Haji Abdul Majid, Mahmoud Amreia, Dina Al-Marakby, Mahmoud Salma, Mohamad Jeffrey Bin Ismail, Elissa Rifhan Mohd Basir, Citra Dewi Mohd Ali, Aya Yehia Ata (Faculty Of Medicine, Tanta University); Maha Nasr, Asmaa Rezq, Ahmed Sheta, Sherif Tariq, Abd Elkhalek Sallam, Abdelrhman KZ Darwish, Sohaila Elmihy, Shady Elhadry, Ahmed Farag, Haidar Hajeh, Abdelaziz Abdelaal, Amro Aglan, Ahmed Zohair, Mahitab Essam, Omar Moussa, Esraa El-Gizawy, Mostafa Samy, Safia Ali, Esraa Elhalawany, Ahmed Ata, Mohamed El Halawany, Mohamed Nashat, Samar Soliman, Alaa Elazab, Mostada Samy, (El-Menshawy General Hospital); Mohamed A Abdelaziz, Khaled Ibrahim, Ahmed mohamed Ibrahim, Ammar Gado, Usama Hantour (Al-Hussein Hospital); Esraa Alm Eldeen, Mohamed Reda loaloa, Arwa Abouzaid, Mostafa Ahmed Bahaa Eldin, Eman Hashad, Fathy Sroor, Doaa Gamil, Eman Mahmoud Abdulhakeem, Mahmoud Zakaria, Fawzy Mohamed, Marwan Abubakr, Elsayed Ali, Hesham Magdy, Menna Tallah Ramadan, Mohamed Abdelaty Mohamed, Salma Mansour, Hager Abdul Aziz Amin, Ahmed Rabie Mohamed, Mahmoud Saami, Nada Ahmed Reda Elsayed, Adham Tarek, Sabry Mohy Eldeen Mahmoud, Islam Magdy El Sayed, Amira Reda, Martina Yusuf Shawky, Mohammed Mousa Salem, Shahinaz Alaa El-Din, Noha Abdullah Soliman, Muhammed Talaat, Shahinaz Alaael-Dein, Ahmed Abd Elmoen Elhusseiny, Noha Abdullah, Mohammed Elshaar, Aya AbdelFatah Ibraheem, Hager Abdulaziz, Mohammed Kamal Ismail, Mona Hamdy Madkor, Mohamed Abdelaty, Sara Mahmoud Abdel-Kader, Osama Mohamed Salah, (Benha Faculty Of Medicine); Mahmoud Eldafrawy, Ahmed Zaki Eldeeb, Mostafa Mahmoud Eid (October 6 University Hospital); Attia Attia, Khalid Salah El-Dien, Ayman Shwky (Bab El-Shareia University Hospital); Mohamed Adel Badenjki, Abdelrahman Soliman, Samaa Mahmoud Al Attar, (The Memorial Soaad Kafafi University Hospital); Farrag Sayed, Fahd Abdel Sabour, Mohammed G. Azizeldine, Muhammad Shawqi, Abdullah Hashim, Ahmed Aamer, Ahmed Mahmoud Abdelraouf, Mahmoud Abdelshakour, Amal Ibrahim, Basma Mahmoud, Mohamed Ali Mahmoud, Mostafa Qenawy, Ahmed M. Rashed, Ahmed Dahy, Marwa Sayed, Ahmed W. Shamsedine, Bakeer Mohamed, Ahmad Hasan, Mahmoud M. Saad, Khalil Abdul Bassit (Assiut University Hospital); Nadia Khalid Abd El-Latif, Nada Elzahed, Ahmed El Kashash, Nada Mohamed Bekhet, Sarah Hafez, Ahmed Gad, Mahmoud Elkhadragy Maher, Ahmed Abd Elsameea, Mohamed Hafez, Ahmad Sabe, Ataa Ahmed, Ahmed Shahine, Khaled Dawood, Shireen Gaafar, Reem Husseiny, Omnia Aboelmagd, Ahmed Soliman, Nourhan Mesbah, Hossam Emadeldin, Amgad Al Meligy, Amira Hassan Bekhet, Doaa Hasan, Khaled Alhady, Ahmad Khaled Sabe, Mahmoud A. Elnajjar, Majed Aboelella, Ward Hamsho, Ihab Hassan, Hala Saad, Galaleldin Abdelazim, Hend Mahmoud, Noha Wael, Ahmedali M Kandil, Ahmed Magdy, Shimaa Said Elkholy, Badr Eldin Adel, Kareem Dabbour, Saged Elsherbiney, Omar Mattar, Abdulshafi Khaled Abdrabou, Mohammed Yahia Mohamed Aly, Abdelrahman Geuoshy, Ahmedglal Elnagar, Saraibrahim Ahmed, Ibrahem Abdelmotaleb, Amr Ahmed Saleh, Manar Saeed, Shady Mahmoud, Badreldin Adel Tawfik, Samar Adel Ismail, Esraay Zakaria, Mariam O. Gad‏, Mohamed Salah Elhelbawy, Monica Bassem, Noha Maraie, Nourhan Medhat Elhadary, Nourhan Semeda, Shaza Rabie Mohamed, Hesham Mohammed Bakry, AA Essam (Kasr Al-Ainy Faculty Of Medicine, Cairo University); Dina Tarek, Khlood Ashour, Alaa Elhadad, Abdulrahman Abdel-Aty, Ibrahim Rakha, Sara Mamdouh Matter, Rasha Abdelhamed, Omar Abdelkader, Ayat Hassaan, Yasmin Soliman, Amna Mohamed, Sara Ghanem, Sara Amr Mohamed Farouk, Eman Mohamed Ibrahim, Esraa El-Taher (Faculty Of Medicine Seuz Canal University); Merna Mostafa, Mohamed Fawzy Mahrous Badr, Rofida Elsemelawy, Aya El-Sawy, Ahmad Bakr, Ahmad Abdel Razaq Al Rafati (Smouha University Hospital);

**Estonia**: Sten Saar, Arvo Reinsoo, Peep Talving (The North Estonia Medical Centre).

**Ethiopia**: Nebyou Seyoum, Tewodros Worku, Agazi Fitsum, (Addis Ababa University, College of Health Sciences, School of Medicine).

**Finland:** Matti Tolonen, Ari Leppäniemi, Ville Sallinen (Helsinki University Hospital).

**France**: Benoît Parmentier, Matthieu Peycelon, Sabine Irtan (Trousseau Hospital, APHP); Sabrina Dardenne, Elsa Robert (GHICL); Betty Maillot, Etienne Courboin, Alexis Pierre Arnaud, Juliette Hascoet (CHU Rennes); Olivier Abbo, Amir Ait Kaci, Thomas Prudhomme (CHU Toulouse); Quentin Ballouhey, Céline Grosos, Laurent Fourcade (CHU Limoges); Tolg Cecilia, Colombani Jean-Francois, Francois-Coridon Helene (CHU Martinique); Xavier Delforge, Elodie Haraux (CHU Amiens Picardie); Bertrand Dousset, Roberto Schiavone, Sebastien Gaujoux, (Cochin - APHP); Jean-Baptiste Marret, Aurore Haffreingue, Julien Rod (CHU Caen); Mariette Renaux-Petel (CHU Rouen); Jean-François Lecompte, Jean Bréaud, Pauline Gastaldi (CHU Lenval Nice); Chouikh Taieb, Raquillet Claire, Echaieb Anis (Hopital Robert Ballanger, Paris); Nasir Bustangi, Manuel Lopez, Aurelien Scalabre (CHU De Saint Etienne); Maria Giovanna Grella (CHU Poitiers); Aurora Mariani, Guillaume Podevin, Françoise Schmitt (CHU Angers); Erik Hervieux, Aline Broch, Cecile Muller (Hopital Necker Enfants Malades, APHP).

**Ghana**: Stephen Tabiri, Anyomih Theophilus Teddy Kojo, Dickson Bandoh, Francis Abantanga, Martin Kyereh, Hamza Asumah, Eric Kofi Appiah, Paul Wondoh (Tamale Teaching Hospital); Adam Gyedu, Charles Dally, Kwabena Agbedinu, Michael Amoah, Abiboye Yifieyeh, (Kwame Nkrumah University of Science and Technology/Komfo Anokye Teaching Hospital); Frank Owusu (St. Particks Hospital); Mabel Amoako-Boateng, Makafui Dayie, Richmond Hagan, Sam Debrah, (Cape Coast Teaching Hosital); Micheal Ohene-Yeboah, Joe-Nat Clegg-Lampety, Victor Etwire, Jonathan Dakubo, Samuel Essoun, William Bonney, Hope Glover-Addy, Samuel Osei-Nketiah, Joachim Amoako, Niiarmah Adu-Aryee, William Appeadu-Mensah, Antoinette Bediako-Bowan, Florence Dedey (Korle Bu Teaching Hospital); Mattew Ekow, Emmanuel Akatibo, Musah Yakubu (Baptist Medical Center, Nalerigu); Hope Edem Kofi Kordorwu, Kwasi Asare-Bediako, Enoch Tackie (Keta Hospital District Hospital, Keta); Kenneth Aaniana, Emmanuel Acquah, Richard Opoku-Agyeman, Anthony Avoka, Kwasi Kusi, Kwame Maison, (Techiman Holy Family Hospital); Frank Enoch Gyamfi (Berekum Holy Family Hospital); Gandau Naa Barnabas, Saiba Abdul-Latif (Upper West Regional Hospital); Philip Taah Amoako (Samapa Government Hospital); Anthony Davor, Victor Dassah (Upper East Regional Hospital); Enoch Dagoe (St. Mary's Hospital); Prince Kwakyeafriyie (Essumejaman Sda Hospital, Dominase); Elliot Akoto, Eric Ackom, Ekow Mensah (Dormaa Presbyterian Hospital); Ebenezer Takyi Atkins, Christian Lari Coompson (Brongho-Ahafo Regional Hospital, Sunyani)

**Greece:** Nikolaos Ivros, Christoforos Ferousis, Vasileios Kalles, Christos Agalianos, Ioannis Kyriazanos, Christos Barkolias, Angelos Tselos, Georgios Tzikos, Evangelos Voulgaris (Naval And Veterans Hospital); Dimitrios Lytras, Athanasia Bamicha, Kyriakos Psarianos (Achillopoyleio General Hospital Of Volos); Anastasios Stefanopoulos (General Hospital Of Nafplio, Department Of Surgery); Ioannis Patoulias, Dimitrios Sfougaris, Ioannis Valioulis (G. Gennimatas Hospital); Dimitrios Balalis, Dimitrios Korkolis, Dimitrios K Manatakis (Saint Savvas Cancer Hospital); Georgios Kyrou, Georgios Karabelias, Iason-Antonios Papaskarlatos (General And Oncological Hospital Of Kifissia-Athens); Kolonia Konstantina, Nikolaos Zampitis, Stylianos Germanos (General Hospital Of Larissa); Aspasia Papailia, Theodosios Theodosopoulos, Georgios Gkiokas (2nd Dept Of Surgery, Aretaieion Hospital, National & Kapodistrian University Of Athens School Of Medicine); Magdalini Mitroudi, Christina Panteli, Thomas Feidantsis, Konstantinos Farmakis, (G. Gennimatas General Hospital); Dimitrios Kyziridis, Orestis Ioannidis, Styliani Parpoudi (4th Surgical Department, Aristotle University Of Thessaloniki, General Hospital, Papanikolaou); Georgios Gemenetzis, Stavros Parasyris (Attikon University Hospital); Christos Anthoulakis, Nikolaos Nikoloudis, Michail Margaritis (Serres General Hospital);

**Guatemala:** Maria-Lorena Aguilera-Arevalo, Otto Coyoy-Gaitan, Javier Rosales (Hospital General San Juan De Dios); Luis Tale, Rafael Soley, Emmanuel Barrios (Juan Josè Arevalo Bermejo); Servio Tulio Torres Rodriguez, Carlos Paz Galvez, Danilo Herrera Cruz (Hospital San Vicente); Guillermo Sanchez Rosenberg, Alejandro Matheu, David Monterroso Cohen (Hospital Herrera Llerandi).

**Haiti:** Marie Paul, Angeline Charles (Hopital Universitaire De Mirebalais).

**Hong Kong SAR, China:** Justin Chak Yiu Lam, Man Hon Andrew Yeung, Chi Ying Jacquelyn Fok, Ka Hin Gabriel Li, Anthony Chuk-Him Lai, Yuk Hong Eric Cheung, Hong Yee Wong, Ka Wai Leung, Tien Seng Bryan Lee, Wai Him Lam, Weihei Dao, Stephanie Hiu-wai Kwok, Tsz-Yan Katie Chan, Yung Kok Ng, TWC Mak (Prince of Wales Hospital); Qinyang Liu, Chi Chung Foo, James Yang, (University of Hong Kong).

**India:** Basant Kumar, Ankur Bhatnagar, Vijaid Upadhyaya, (Sanjay Gandhi Post Graduate Institute of Medical Sciences); Sunil Kumar (Excelcare Hospital); Uday Muddebihal, Wasim Dar, KC Janardha (Manipal Hospital); Philip Alexander, Neerav Aruldas, (Lady Willingdon Hospital).

**Indonesia:** Fidelis Jacklyn Adella, Anthonius Santoso Rulie, Ferdy Iskandar, Jonny Setiawan (Atma Jaya Hospital); Cicilia Viany Evajelista, Hani Natalie, Arlindawati Suyadi (Dr. Oen Surakarta Hospital); Rudy Gunawan, Herlin Karismaningtyas, Lusi Padma Sulistianingsih Mata, Ferry Fitriya Ayu Andika, Afifatun Hasanah, T Ariani Widiastini, Nurlaila Ayu Purwaningsih, Annisa Dewi Fitriana Mukin, Dina Faizatur Rahmah, Hazmi Dwinanda Nurqistan, Hasbi Maulana Arsyad, Novia Adhitama (Rsd Dr Soebandi); Wifanto Saditya Jeo, Nathania Sutandi, Audrey Clarissa, Phebe Anggita Gultom, Matthew Billy, Andreass Haloho, Radhian Amandito, Nadya Johanna, Felix Lee (Rsupn Cipto Mangunkusumo);

**Ireland:** Radin Mohd Nurrahman Radin Dorani, Martha Glynn, Mohammad Alherz, Wennweoi Goh, Haaris A. Shiwani, Lorraine Sproule, Kevin C. Conlon (Tallaght Hospital, Trinity College Dublin)

**Israel:** Miklosh Bala, Asaf Kedar (Hadassah Hebrew University Medical Center).

**Italy:** Luca Turati, Federica Bianco, Francesca Steccanella, (Treviglio Hospital); Gaetano Gallo, Mario Trompetto, Giuseppe Clerico (Department of Colorectal Surgery, S. Rita Clinic, Vercelli); Matteo Papandrea, Giuseppe Sammarco, Rosario Sacco (Department of Medical and Surgical Sciences, Policlinico Universitario Mater Domini Campus Salvatore Venuta, Catanzaro); Angelo Benevento, Francesco Pata, Luisa Giavarini (Sant'Antonio Abate Hospital, Gallarate); Mariano Cesare Giglio, Luigi Bucci, Gianluca Pagano, Viviana Sollazzo, Roberto Peltrini, Gaetano Luglio (Federico II University Of Naples); Arianna Birindelli, Salomone Di Saverio, Gregorio Tugnoli (Maggiore Hospital); Miguel Angel Paludi, Pietro Mingrone, Domenica Pata (Nicola Giannettasio Hospital, Rossano); Francesco Selvaggi, Lucio Selvaggi, Gianluca Pellino, Natale Di Martino (Universitá della Campania “Luigi Vanvitelli”, Naples); Gianluca Curletti, Paolo Aonzo, Raffaele Galleano (Ospedale Santa Corona, Pietra Ligure (SV)); Stefano Berti, Elisa Francone, Silvia Boni, (S. Andrea Hospital, Poll-Asl 5, La Spezia), Laura Lorenzon, Annalisa lo Conte, Genoveffa Balducci (Sant’Andrea Hospital, Sapienza University of Rome); Gianmaria Confalonieri, Giovanni Pesenti (Azienda Ospedaliera Alessandro Manzoni); Laura Gavagna, Giorgio Vasquez, Simone Targa, Savino Occhionorelli, Dario Andreotti (Azienda Ospedaliero-Universitaria Di Ferrara); Giacomo Pata, Andrea Armellini, Deborah Chiesa (A.O. Spedali Civili Di Brescia); Fabrizio Aquilino, Nicola Chetta, Arcangelo Picciariello (Azienda Ospedaliero Universitaria Consorziale Policlinico Di Bari); Mohamed Abdelkhalek, Andrea Belli, Silvia De Franciscis (Istituto Nazionale Tumori Fondazione, Pascale-I.R.C.C.S.); Annamaria Bigaran, Alessandro Favero, Stefano M.M Basso (Azienda Per L'assistenza Sanitaria N. 5 Friuli Occidentale); Paola Salusso, Martina Perino, Sylvie Mochet, Diego Sasia, Francesco Riente, Marco Migliore (Azienda Sanitaria Ospedaliera San Luigi Gonzaga); David Merlini, Silvia Basilicò, Carlo Corbellini (Ospedale Di Rho - ASST Rhodense); Veronica Lazzari, Yuri Macchitella, Luigi Bonavina (IRCCS Policlinico, University of Milano, San Donato); Daniele Angelieri, Diego Coletta, Federica Falaschi, Marco Catani, Claudia Reali, Mariastella Malavenda, Celeste Del Basso, Sergio Ribaldi, Massimo Coletti, Andrea Natili, Norma Depalma, Immacolata Iannone, Angelo Antoniozzi, Davide Rossi (Policlinico Umberto I Emergency Surgery Department); Daniele Gui, Gerardo Perrotta, Matteo Ripa, Francesco Ruben Giardino, Maurizio Foco, (Fondazione Policlinico Universitario Agostino Gemelli); Erika Vicario, Federico Coccolini, Luca Ansaloni, Gabriela Elisa Nita (AO Papa Giovanni XXIII); Nicoletta Leone, Andrea Bondurri, Anna Maffioli (Ospedale Sacco); Andrea Simioni, Davide De Boni, Sandro Pasquali (IOV - Istituto Oncologico Veneto); Elena Goldin, Elena Vendramin, Eleonora Ciccioli (Azienda Ospedaliera di Padova); Umberto Tedeschi, Luca Bortolasi, Paola Violi, Tommaso Campagnaro, Simone Conci, Giovanni Lazzari, Calogero Iacono, Alfredo Gulielmi, Serena Manfreda (Azienda Ospedaliera Universitaria Integrata di Verona); Anna Rinaldi, Maria Novella Ringressi, Beatrice Brunoni (Azienda Ospedaliera Universitaria Careggi); Giuseppe Salamone, Mirko Mangiapane, Paolino De Marco, Antonella La Brocca, Roberta Tutino, Vania Silvestri, Leo Licari, Tommaso Fontana, Nicolò Falco, Gianfranco Cocorullo, (Policlinico Paolo Giaccone di Palermo); Mostafa Shalaby, Pierpaolo Sileri, Claudio Arcudi (Policlinico Tor Vergata Hospital, Rome)

**Jordan:** Isam Bsisu, Khaled Aljboor, Lana Abusalem, Aseel Alnusairat, Ahmad Qaissieh, Emad Al-Dakka, Ali Ababneh, Oday Halhouli (Jordan University Hospital).

**Kenya:** Taha Yusufali, Hussein Mohammed (Kenyatta National Hospital); Justus Lando, Robert Parker, Wairimu Ndegwa (Tenwek Hospital).

**Lithuania:** Mantas Jokubauskas, Jolanta Gribauskaite, Donatas Venskutonis (Lithuanian University Of Health Sciences); Justas Kuliavas, Audrius Dulskas, Narimantas E. Samalavicius (Klaipeda University Hospital, National Cancer Institute); Kristijonas Jasaitis, Audrius Parseliunas, Viktorija Nevieraite, Margarita Montrimaite, Evelina Slapelyte, Edvinas Dainius, Romualdas Riauka, Zilvinas Dambrauskas, Andrejus Subocius, Linas Venclauskas, Antanas Gulbinas, Saulius Bradulskis, Simona Kasputyte, Deimante Mikuckyte, Mindaugas Kiudelis, Justas Zilinskas, Tomas Jankus, Steponas Petrikenas (Lithuanian University of Health Sciences); Matas Pažuskis, Zigmantas Urniežius, Mantas Vilčinskas (Republican Hospital of Kaunas); Vincas Jonas Banaitis, Vytautas Gaižauskas, Edvard Grisin, Povilas Mazrimas, Rokas Rackauskas, Mantas Drungilas, Karolis Lagunavicius, Vytautas Lipnickas, Dovilè Majauskyté, Valdemaras Jotautas, Tomas Abaliksta, Laimonas Uščinas, Gintaras Simutis, Adomas Ladukas, Donatas Danys, Erikas Laugzemys, Saulius Mikalauskas, Tomas Poskus, Elena Zdanyte Sruogiene, (Vilnius University Hospital); Petras Višinskas, Reda Žilinskienė, Deividas Dragatas (Hospital Of Jonava); Andrius Burmistrovas, Zygimantas Tverskis (Taurage Hospital); Arturas Vaicius, Ruta Mazelyte, Antanas Zadoroznas (Viesoji Istaiga Rokiskio Rajono Ligonine); Nerijus Kaselis, Greta Žiubrytė, (Republican Hospital Of Klaipeda);

**Madagascar:** Finaritra Casimir Fleur Prudence Rahantasoa, Luc Hervé Samison, Fanjandrainy Rasoaherinomenjanahary, Todisoa Emmanuella Christina Tolotra (Joseph Ravoahangy Andrianavalona Hospital).

**Malawi:** Cornelius Mukuzunga, Vanessa Msosa, Chimwemwe Kwatiwani, Nelson Msiska (Kamuzu Central Hospital).

**Malaysia:** Feng Yih Chai, Siti Mohd Desa Asilah, Khuzaimah Zahid Syibrah (Hospital Keningau); Pui Xin Chin, Afizah Salleh, Nur Zulaika Riswan (Kajang Hospital); April Camilla Roslani, Hoong-Yin Chong, Nora Abdul Aziz, Keat-Seong Poh, Chu-Ann Chai, Sandip Kumar (University Malaya Medical Centre); Mustafa Mohammed Taher, Nik Ritza Kosai, Dayang Nita Abdul Aziz, Reynu Rajan (Universiti Kebangsaan Malaysia Medical Centre UKMMC); Rokayah Julaihi, Durvesh Lacthman Jethwani, Muhammad Taqiyuddin Yahaya, Nik Azim Nik Abdullah, Susan Wndy Mathew, Kuet Jun Chung, Milaksh Kumar Nirumal, R. Goh Ern Tze, Syed Abdul Wahhab Eusoffee Wan Ali (Sarawak General Hospital); Yiing Yee Gan, Jesse Ron Swire Ting (Hospital Sibu); Samuel S. Y. Sii, Kean Leong Koay, Yi Koon Tan, Alvin Ee Zhiun Cheah, Chui Yee Wong, Tuan Nur'Azmah Tuan Mat, Crystal Yern Nee Chow, Prisca A.L. Har, Yishan Der (Hospital Sultanah Aminah); Yong Yong Tew, Fitjerald Henry, Xinwei Low (Selayang Hospital); Ya Theng Neo, Hian Ee Heng, Shu Ning Kong, Cheewei Gan, Yi Ting Mok, Yee Wen Tan, Kandasami Palayan, Mahadevan Deva Tata, Yih Jeng Cheong (Hospital Tuanku Ja'afar); Kuhaendran Gunaseelan, Wan Nurul 'Ain Wan Mohd Nasir, Pigeneswaren Yoganathan, (Hospital Keningau); Eu Xian Lee, Jian Er Saw, Li Jing Yeang, Pei Ying Koh, Shyang Yee Lim, Shuang Yi Teo (Hospital Pulau Pinang / Penang Medical College);

**Malta:** Nicole Grech, Daniela Magri, Kristina Cassar, Christine Mizzi, Malcolm Falzon, Nihaal Shaikh, Ruth Scicluna, Stefan Zammit, Elaine Borg, Sean Mizzi, Svetlana Doris Brincat, Thelma Tembo, Vu Thanh Hien Le, Tara Grima, Keith Sammut, Kurt Carabott, Alexia Farrugia, Ciskje Zarb, Andre Navarro, Thea Dimech, Georgette Marie Camilleri, Isaac Bertuello, Jeffrey Dalli, Karl Bonavia (Mater Dei Hospital).

**Mexico**: Samantha Corro-Diaz, Marisol Manriquez-Reyes, Antonio Ramos-De la Medina (Hospital Español de Veracruz).

**Morocco**: Amina Abdelhamid, Abdelmalek Hrora, Sarah Benammi, Houda Bachri, Meryem Abbouch, Khaoula Boukhal, Redouane Mammar Bennai, Abdelkader Belkouchi, Mohamed Sobhi Jabal, Chaymae Benyaiche (IBN Sina Hospital)

**Netherlands:** Maarten Vermaas, Lucia Duinhouwer, (Ijsselland Hospital)

**Nicaragua:** Javier Pastora, Greta Wood, Maria Soledad Merlo (Hospital Escuela Oscar Danilo Rosales Arguello).

**Nigeria:** Akinlabi Ajao, Omobolaji Ayandipo, Taiwo Lawal, Abdussemiu Abdurrazzaaq, (University College Hospital, Ibadan); Muslimat Alada, Abdulrasheed Nasir, James Adeniran, Olufemi Habeeb, Ademola Popoola, Ademola Adeyeye (University Of Ilorin Teaching Hospital,Ilorin); Ademola Adebanjo, Opeoluwa Adesanya, Adewale Adeniyi (Federal Medical Centre, Abeokuta,); Henry Mendel, Bashir Bello, Umar Muktar (Usmanu Danfodiyo University Teaching Hospital);); Adedapo Osinowo, Thomas Olagboyega Olajide, Oyindamola Oshati, George Ihediwa, Babajide Adenekan, Victor Nwinee, Felix Alakaloko, Adesoji Ademuyiwa, Olumide Elebute, Abdulrazzaq Lawal, Chris Bode, Mojolaoluwa Olugbemi (Lagos University Teaching Hospital); Alaba Adesina, Olubukola Faturoti, Oluwatomi Odutola, Oluwaseyi Adebola, Clement Onuoha, Ogechukwu Taiwo (Babcock University Teaching Hospital); Omolara Williams, Fatai Balogun, Olalekan Ajai, Mobolaji Oludara, Iloba Njokanma, Roland Osuoji (Lagos State University Teaching Hospital); Stephen Kache, Jonathan Ajah, Jerry Makama (Barau Dikko Teaching Hospital, Kaduna State University, Kaduna); Ahmed Adamu, Suleiman Baba, Mohammad Aliyu, Shamsudeen Aliyu, Yahaya Ukwenya, Halima Aliyu, Tunde Sholadoye, Muhammad Daniyan, Oluseyi Ogunsua (Ahmadu Bello University Teaching Hospital Zaria); Lofty-John Anyanwu, Abdurrahaman Sheshe, Aminu Mohammad (Aminu Kano Teaching Hospital); Samson Olori, Philip Mshelbwala, Babatunde Odeyemi, Garba Samson, Oyediran Kehinde Timothy, Sani Ali Samuel (University Of Abuja Teaching Hospital,); Anthony Ajiboye, Ademola Adeyeye, Isaac Amole, Olajide Abiola, Akin Olaolorun (Bowen University Teaching Hospital);

**Norway:** Kjetil Søreide, Torhild Veen, Arezo Kanani, Kristian Styles, Ragnar Herikstad, Johannes Wiik Larsen, Jon Arne Søreide (Stavanger University Hospital); Elisabeth Jensen, Mads Gran, Eirik Kjus Aahlin (University Hospital Of Northern Norway); Tina Gaarder, Peter Wiel Monrad-Hansen, Pål Aksel Næss (Oslo University Hospital); Giedrius Lauzikas, Joachim Wiborg, Silje Holte (Sykehuset Telemark HF); Knut Magne Augestad, Gurpreet Singh Banipal, Michela Monteleone, Thomas Tetens Moe, Johannes Kurt Schultz (Akershus University Hospital);

**Palestine:** Taher Al-taher, Ayah Hamdan, Ayman Salman, Rana Saadeh, Aseel Musleh, Dana Jaradat, Soha Abushamleh, Sakhaa Hanoun, Amjad Abu Qumbos, Aseel Hamarshi, Ayman And Taher (Al Makassed Islamic Charitable Society Hospital, Jerusalem); Israa Qawasmi, Khalid Qurie, Marwa Altarayra, Mohammad Ghannam, Alaa Shaheen, Azher Herebat (Alia Governmental Hospital); Aram Abdelhaq, Ahmad Shalabi, Maram Abu-toyour, Fatema Asi, Ala Shamasneh, Anwar Atiyeh, Mousa Mustafa, Rula Zaa'treh, Majd Dabboor (Palestine Medical Complex); Enas Alaloul, Heba Baraka, Jehad Meqbil, Alaa Al-Buhaisi, Mohamedraed Elshami, Samah Afana, Sahar Jaber, Said Alyacoubi, Yousef Abuowda (Islamic University of Gaza Medical School, European Gaza Hospital & Shifa Hospital); Tasneem Idress, Eman Abuqwaider (Mizan hospital); Sara Al-saqqa, Alaa Bowabsak, Alaa El Jamassi, Doaa Hasanain, Hadeel Al-farram, Maram Salah, Aya Firwana, Marwa Hamdan, Israa Awad (Al-Shifa Hospital); Ahmad Ashour, Fayez Elian Al Barrawi (Bit Hanoun Hospital); Ahmed Al-khatib, Maha Al-faqawi, Mohamed Fares (Nasser Hospital); Amjad Elmashala, Mohammad Adawi, Ihdaa Adawi (Beit Jala Governmental Hospital); Reem Khreishi, Rose Khreishi (Martyr Thabet Govermental hospital, Tulkarem); Ahmad ashour, Ahed Ghaben (Indonesian Hospital, Gaza)

**Pakistan:** Najwa Nadeem, Muhammad Saqlain (Allied Hospital, Faisalabad); Jibran Abbasy, Abdul Rehman Alvi, Tanzeela Gala, Noman Shahzad (Aga Khan University); Kamran Faisal Bhopal, Zainab Iftikhar, Muhammad Talha Butt, Syed Asaat ul Razi, Asdaq Ahmed, Ali Khan Niazi (Bahawal Victoria Hospital); Ibrahim Raza, Fatima Baluch, Ahmed Raza, Ahmad Bani-Sadar, Ahmad Uzair Qureshi, Muhammad Adil, Awais Raza, (King Edward Medical University, Mayo Hospital, Lahore); Mahnoor Javaid, Muhammad Waqar, Maryam Ali Khan (CMH Lahore Medical And Dental College); Mohammad Mohsin Arshad, Mohammadasim Amjad (Nishtar Medical College And Hospital);

**Paraguay:** Gustavo Miguel Machain Vega, Jorge Torres Cardozo, Marcelo O´Higgins Roche, Gustavo Rodolfo Pertersen Servin, Helmut Alfredo Segovia Lohse, Larissa Ines Páez Lopez, Ramón Augusto Melo Cardozo (Hospital de Clínicas, II Cátedra de Clínica Quirúrgica, Universidad Nacional de Asunción)

**Peru:** Fernando Espinoza, Angel David Pérez Rojas, Diana Sanchez, Camila Sanchez Samaniego, Shalon Guevara Torres, Alexander Canta Calua, Cesar Razuri, Nadia Ortiz, Xianelle Rodriguez, Nahilia Carrasco, Fridiz Saravia, Hector Shibao Miyasato, María Valcarcel-Saldaña, Ysabel Esthefany Alejos Bermúdez, Juan Carpio, Walter Ruiz Panez, Pedro Angel Toribio Orbegozo, (Hospital Nacional Arzopispo Loayza); Carolina Guzmán Dueñas, Kevin Turpo Espinoza, Ana Maria Sandoval Barrantes, Jorge Armando Chungui Bravo, Sebastian Shu, Lorena Fuentes-Rivera, Carmen Fernández, Diego Romani, Bárbara Málaga, Joselyn Ye (Hospital Cayetano Heredia); Ricardo Velasquez, Jannin Salcedo (Clínica De Especialidades Médicas); Ana Lucia Contreras-Vergara, Angelica Genoveva Vergara Mejia, Maria Soledad Gonzales Montejo (Hospital Nacional Guillermo Almenara Irigoyen); Marilia Del Carmen Escalante Salas, Willy Alcca Ticona, Marvin Vargas, George Christian Manrique Sila, Robinson Mas, Arazzelly del Pilar Paucar (Hospital Regional De Ayacucho); Armando José Román Velásquez, Alina Robledo-Rabanal, Ludwing Alexander Zeta Solis, (Hospital III José Cayetano Heredia); Kenny Turpo Espinoza (Hospital Nacional Maria Auxiliadora); José Luis Hamasaki Hamaguchi, Erick Samuel Florez Farfan, Linda Alvi Madrid Barrientos, Juan Jaime Herrera Matta (Hospital De Policia);

**Philippines:** John Jemuel V. Mora, Menold Archee P. Redota, Manuel Francisco Roxas, Maria Jesusa B. Maño, (The Medical City); Marie Dione Parreno-Sacdalan, Marie Carmela Lapitan, Christel Leanne Almanon (Department of Surgery, Philippine General Hospital, University of The Philippines Manila).

**Poland:** Maciej Walędziak, Rafał Roszkowski, Michał Janik (Department of General, Oncological, Metabolic and Thoracic Surgery, Military Institute of Medicine, Warsaw); Anna Lasek, Piotr Major, Dorota Radkowiak, Mateusz Rubinkiewicz (2nd Department of Surgery, Jagiellonian University Medical College).

**Portugal:** Cristina Fernandes, Jose Costa-Maia, Renato Melo (Centro Hospitalar De São João).

**Romania:** Liviu Muntean, Aurel Sandu Mironescu, Lucian Corneliu Vida (Spitalul Clinic De Copii Brasov); Amar Kourdouli, Mariuca Popa (Spital Judetean De Urgenta Din Craiova); Hogea Mircea (Spitalul Clinic Judetean Brasov); Mihaela Vartic, Bogdan Diaconescu, Matei Razvan Bratu, Ionut Negoi, Mircea Beuran, Cezar Ciubotaru (Emergency Hospital of Bucharest).

**Rwanda:** J.C Allen Ingabire, Alphonse Zeta Mutabazi, Norbert Uzabumwana (University Teaching Hospital of Kigali); Dieudonne Duhoranenayo (Kibungo Hospital).

**San Marino:** Elio Jovine, Nicola Zanini, Giovanni Landolfo (San Marino State Hospital).

**Saudi Arabia:** Murad Aljiffry, Faisal Idris, Mohammed Saleh A. Alghamdi, Ashraf Maghrabi, Abdulmalik Altaf, Aroub Alkaaki, Ahmad Khoja, Abrar Nawawi, Sondos Turkustani, (Department of Surgery, Faculty of Medicine, King Abdulaziz University Hospital, Jeddah) Eyad Khalifah, Ahmad Gudal, Adel Albiety, Sarah Sahel, Reham Alshareef, Mohammed Najjar (Department of Surgery, King Abdulaziz University Hospital and Oncology Center, Jeddah) Ahmed Alzahrani, Ahmed Alghamdi, Wedyan Alhazmi, (King Fahad Hospital, Jeddah) Ghiath Al Saied, Mohammed Alamoudi, Muhammed Masood Riaz (King Fahad Medical City, Riyadh); Mazen Hassanain, Basmah Alhassan, Abdullah Altamimi, Reem Alyahya, Norah Al Subaie, Fatema Al Bastawis, Afnan Altamimi, Thamer Nouh, Roaa Khan, (King Khaled University Hospital);

**Serbia:** Milan Radojkovic, Ljiljana Jeremic, Milica Nestorovic (Clinic for General Surgery, Clinical Center Nis).

**Singapore:** Jia Hao Law, Keith Say Kwang Tan, Ryan Choon Kiat Tan, Joel Kin Tan, Lau Wen Liang Joel, Bettina Lieske, Xue Wei Chan, Faith Qi Hui Leong, Choon Seng Chong, Sharon Koh, Kai Yin Lee, Kuok Chung Lee (National University Hospital)

**South Africa:** Kent Pluke, Britta Dedekind, Puyearashid Nashidengo, Mark Ian Hampton (Victoria Hospital Wynberg); Johanna Joosten, Sanju Sobnach, Liana Roodt, Anthony Sander, James Pape, Richard Spence (Groote Schuur); Niveshni Maistry (Charlotte Maxeke Johannesburg Academic Hospital); Phumudzo Ndwambi, Kamau Kinandu, Myint Tun (Leratong Hospital); Frederick Du Toit, Quinn Ellison, Sule Burger, DC Grobler, Lawrence Bongani Khulu (Tembisa Tertiary Provincial Hospital); Rachel Moore, Vicky Jennings, Astrid Leusink (Chris Hani Baragwanath Academic Hospital); Nazmie Kariem, Juan Gouws, Kathryn Chu, Heather Bougard, Fazlin Noor, Angela Dell (New Somerset Hospital); Sarah Rayne, Stephanie Van Straten, (Helen Joseph Hospital, University Of Witwatersrand); Arvin Khamajeet, Serge Kapenda Tshisola, Kalangu Kabongo (Stanger Hospital); Victor Kong (Edendale Hospital); Yoshan Moodley, Frank Anderson, Thandinkosi Madiba (Inkosi Albert Luthuli Central Hospital); Flip du Plooy (Mediclinic Potchefstroom); Leila Hartford, Gareth Chilton, Parveen Karjiker (Mitchell's Plain District Hospital); Matlou Ernest Mabitsela, Sibongile Ruth Ndlovu (Dr George Mukhari Academic Hospital); Maria Badicel, Robert Jaich (Milpark Hospital)

**Spain:** Jaime Ruiz-Tovar (University Hospital Rey Juan Carlos); Luis Garcia-Florez, Jorge L. Otero-Díez, Virginia Ramos Pérez, Nuria Aguado Suárez (Hospital Universitario San Agustín); Javier Minguez García, Sara Corral Moreno, Maria Vicenta Collado , Virginia Jiménez Carneros, Javier García Septiem (Hospital Universitario de Getafe); Mariana Gonzalez, Antonio Picardo, Enrique Esteban, Esther Ferrero, Irene Ortega, (Infanta Sofía University Hospital); Eloy Espin-Basany, Ruth Blanco-Colino, Valeria Andriola (Hospital Valle De Hebron); Lorena Solar García, Elisa Contreras, Carmen García Bernardo, Janet Pagnozzi, Sandra Sanz, Alberto Miyar de León, Asnel Dorismé, Joseluis Rodicio, Aida Suarez, Jessica Stuva, Tamara Diaz Vico (Central University Hospital Of Asturias); Laura Fernandez-Vega, Carla Soldevila-Verdeguer, Fatima Sena-Ruiz, Natalia Pujol-Cano, Paula Diaz-Jover, José Maria Garcia-Perez, Juan Jose Segura-Sampedro, Cristina Pineño-Flores, David Ambrona-Zafra, Andrea Craus-Miguel, Patricia Jimenez-Morillas, Angela Mazzella (Hospital Universitario Son Espases);

**Sri Lanka**: A.B Jayathilake, S.P.B Thalgaspitiya, L.S. Wijayarathna, P.M.S.N. Wimalge (University Surgical Unit, Teaching Hospital Anuradhapura).

**St. Kitts And Nevis:** Hakeem Ayomi Sanni, Aliyu Ndajiwo, Ogheneochuko Okenabirhie (Joseph N France Hospital)

**Sudan:** Anmar Homeida, Abobaker Younis, Omer Abdelbagi Omer, Mustafa Abdulaziz, Ali Mussad, Ali Adam (University of Gezira).

**Sweden:** Yucel Cengiz, Ida Björklund, Sandra Ahlqvist, Sandra Ahlqvist, (Sundsvall Hospital); Anders Thorell, Fredrik Wogensen (Ersta Hospital); Arestis Sokratous, Michaela Breistrand (Mora Hospital); Hildur Thorarinsdottir (Helsingborgs Lasarett); Johanna Sigurdadottir, Maziar Nikberg, Abbas Chabok (Västmanlands Hospital Västerås); Maria Hjertberg (Department Of Surgery And Department Of Clinical And Experimental Medicine, Linköping University, Norrköping, Sweden); Peter Elbe, Deborah Saraste, Wiktor Rutkowski, Louise Forlin (Karolinska Universitetssjukhuset, Solna); Karoliina Niska, Malin Sund (Umea University Hospital)

**Switzerland:** Dennis Oswald, Georgios Peros, Rafael Bluelle, Katharina Reinisch, Daniel Frey, Adrian Palma (Gzo Spital Wetzikon); Dimitri Aristotle Raptis, Lucius Zumbühl, Markus Zuber (Kantonsspital Olten); Roger Schmid, Gabriela Werder (Buergerspital Solothurn); Antonio Nocito, Alexandra Gerosa, Silke Mahanty (Kantonsspital Baden); Lukas Werner Widmer, Julia Müller, Alissa Gübeli (Hospital Davos); Grzegorz Zuk (Gzo Spital Wetzikon).

**Turkey:** Osman Bilgin Gulcicek, Yuksel Altinel, Talar Vartanoglu, (Bagcilar Research And Training Hospital); Emin Kose, Servet Rustu Karahan, Mehmet Can Aydin (Okmeydanı Training And Research Hospital); Nuri Alper Sahbaz, Ilkay Halicioglu, Halil Alis (Bakirkoy Dr. Sadi Konuk Training And Research Hospital); Ipek Sapci, Can Adıyaman, Ahmet Murat Pektaş, Turgut Bora Cengiz, Ilkan Tansoker, Vedatcan Işler, Muazzez Cevik, Deniz Mutlu, Volkan Ozben, Berk Baris Ozmen, Sefa Bayram, Sinem Yolcu, Berna Buse Kobal, Ömer Faruk Toto, Haluk Cem Çakaloğlu (Acibadem University School of Medicine, Atakent Hospital); Kagan Karabulut, Vahit Mutlu, Bahar Busra Ozkan (Ondokuz Mayis University Medical Faculty); Saban Celik, Anil Semiz, Selim Bodur, Enisburak Gül, Busra Murutoglu, Reyyan Yildirim, Bahadir Emre Baki, Ekin Arslan, Mehmet Ulusahin, Ali Guner (Karadeniz Technical University Faculty Of Medicine);

**United Kingdom:** Nathan Walker, Nikhita Shrimanker, Michael Stoddart, Simon Cole (Royal United Hospital Bath); Ryan Breslin, Ravi Srinivasan (Blackpool Victoria Hospital); Mohamed Elshaer, Kristina Hunter, Ahmed Al-Bahrani (Watford General Hospital); Ignatius Liew, Nora Grace Mairs, Alistair Rocke, Lachlan Dick, Mobeen Qureshi (Inverclyde Royal Hospital); Debkumar Chowdhury (University Hospital Ayr); Naomi Wright, Clare Skerritt, Dorothy Kufeji (Guy's And St. Thomas' Hospitals); Adrienne Ho, Tharindra Dissanayake, Athula Tennakoon, Wadah Ali, (Pilgrim Hospital, United Lincolnshire Hospitals NHS Trust); Shujing Jane Lim, Charlene Tan, Stephen O'Neill, Catrin Jones (Victoria Hospital Kirkcaldy); Stephen Knight, Dima Nassif, Abhishek Sharma (Perth Royal Infirmary); Oliver Warren, Rebecca White, Aia Mehdi, Nathan Post, Eliana Kalakouti, Enkhbat Dashnyam, Frederick Stourton (Chelsea And Westminster Hospital); Ioannis Mykoniatis, Chelise Currow, (Northampton General Hospital); Francisca Wong, Ashish Gupta, Veeranna Shatkar (Queen's Hospital, BHR University Hospitals NHS Trust); Joshua Luck, Suraj Kadiwar, Alexander Smedley (North Middlesex University Hospital); Rebecca Wakefield, Philip Herrod, James Blackwell, Jonathan Lund, (Royal Derby Hospital); Fraser Cohen, Ashwath Bandi, Stefano Giuliani (St George's Hospital); Giles Bond-Smith, Theodore Pezas, Neda Farhangmehr, Tomas Urbonas, Miklos Perenyei (John Radcliffe Hospital, Oxford); Philip Ireland, Natalie Blencowe, Kirk Bowling, David Bunting (Gloucestershire Royal Hospital); Lydia Longstaff, Neil Smart, Kenneth Keogh (Royal Devon & Exeter Hospital); Hyunjin Jeon, Muhammad Rafaih Iqbal, Shivun Khosla, Anna Jeffery, James Perera (Maidstone & Tunbridge Wells NHS Trust); Ella Teasdale (Western Isles Hospital); Ahmad Aboelkassem Ibrahem, Tariq Alhammali, Yahya Salama (Kettering General Hospital NHS Trust); Rakan Kabariti, Shaun Oram (Nevill Hall Hospital); Thomas Kidd, Fraser Cullen, Christopher Owen, Michael Wilson, Seehui Chiu, Hannah Sarafilovic, (Ninewells Hospital); Jennifer Ploski, Elizabeth Evans, Athar Abbas, Sylvia Kamya, Norzawani Ishak, Carly Bisset, Cedar Andress, Ye Ru Chin (Royal Alexandra Hospital, Paisley); Priya Patel, David Evans (University Hospital, Wales); Anna Jeffery, James Perera (Maistone and Tunbridge Wells NHS Trust); Aidan Haslegrave, Adam Boggon, Kirsten Laurie, Katie Connor, Thomas Mann (Borders General Hospital); Dmitri Nepogodiev, Anahita Mansuri, Rachel Davies, Ewen Griffiths (University Hospitals Birmingham NHS Trust); Aized Raza Shahbaz, Calvin Eng, Farhat Din, Ariadne L'Heveder, Esther H.G. Park, Ramanish Ravishankar, Kirsten McIntosh, Jih Dar Yau, Luke Chan, Susan McGarvie (Western General Hospital, Edinburgh); Lingshan Tang, Hui Lim, Suhhuey Yap, Jay Park, Zhan Herr Ng, Shahrukh Mirza, Yun Lin Ang, Luke Walls, Ella Teasdale, Chloe Roy, Simon Paterson-Brown, Julian Camilleri-Brennan, Kenneth Mclean, Michelle S D'Souza, Savva Pronin, David Ewart Henshall, Eunice Zuling Ter (Royal Infirmary Of Edinburgh); Dina Fouad, Ashish Minocha (Norfolk And Norwich University Hospital); William English, Catrin Morgan, Dominic Townsend, Laura Maciejec, Shareef Mahdi, Onyinye Akpenyi, Elisabeth Hall, Hanaan Caydiid, Zakaria Rob, Tom Abbott, Hew D Torrance (The Royal London Hospital); Gareth Irwin, Robin Johnston (Ulster Hospital Dundonald); Mohammed Akil Gani, Gianpiero Gravante (Leicester Royal Infirmary); Shivanchan Rajmohan, Kiran Majid, Shiva Dindyal, Christopher Smith (West Middlesex University Hospital); Madanmohan Palliyil, Sanjay Patel, Luke Nicholson, Neil Harvey, Katie Baillie, Sam Shillito, Suzanne Kershaw, Rebecca Bamford, Peter Orton (Stockport NHS Foundation Trust); Elke Reunis, Robert Tyler, Wai Cheong Soon (Good Hope Hospital); Guled M. Jama, Dharminder Dhillon, Khyati Patel (Walsall Manor Hospital, Walsall); Shayanthan Nanthakumaran, Rachel Heard, Kar Yan Chen (Aberdeen Royal Infirmary); Behrad Barmayehvar, Uttaran Datta, Sivesh K Kamarajah, Sharad Karandikar (Heartlands Hospital); Sobhana Iftekhar Tani (Nottingham University Hospital NHS Trust); Eimear Monaghan, Philippa Donnelly, Michael Walker (Raigmore Hospital); Jehangirshaw Parakh, Sarah Blacker, Anil Kaul (Whiston Hospital); Arjun Paramasivan (Darlington Memorial Hospital); Sameh Farag, Ashrafun Nessa, Salwa Awadallah (Worthing Hospital, Western Sussex Hospital NHS Foundation Trust); Jieqi Lim, James Chean Khun Ng (Queen Elizabeth University Hospital, Glasgow);

**United States**: Katherine Gash, Ravi P. Kiran, Alice Murray (New York Presbyterian Hospital / Columbia University Medical Center); Eric Etchill, Mohini Dasari, Juan Puyana (University Of Pittsburgh Medical Center - Presbyterian); Nadeem Haddad, Martin Zielinski, Asad Choudhry (Mayo Clinic); Celeste Caliman, Mieshia Beamon, Therese Duane (John Peter Smith Hospital); Ragavan Narayanan, Mamta Swaroop (Northwestern Memorial Hospital / Northwestern University); Jonathan Myers, Rebecca Deal, Erik Schadde (Rush University Medical Centre); Mark Hemmila, Lena Napolitano, Kathleen To (University Of Michigan Medical Center)

**Zambia**: Alex Makupe, Joseph Musowoya, Mayaba Maimbo (Ndola Central Hospital); Niels Van Der Naald, Dayson Kumwenda, Alex Reece-Smith, Kars Otten, Anna Verbeek, Marloes Prins (St Francis Mission Hospital)

*GlobalSurg Collaborative Data Validators:*

**Argentina**: Alibeth Andres Baquero Suarez (Simplemente Evita), Ruben Balmaceda (Hospital Lagomaggiore).

**Barbados**: Chelsea Deane (Queen Elizabeth Hospital).

**Croatia**: Emilio Dijan (Zadar General Hospital).

**Egypt**: Mahmoud Elfiky (Kasr Al Ainy Faculty of Medicine, Cairo University).

**Finland**: Laura Koskenvuo (Helsinki University Hospital).

**France**: Aurore Thollot (CHU Poitiers), Bernard Limoges (CHU Limoges), Carmen Capito (Hopital Necker Enfants Malades, APHP), Challine Alexandre (Hopital Cochin, APHP), Henri Kotobi (Trousseau Hospital, APHP), Julien Leroux (CHU Rouen), Julien Rod (CHU Caen), Kalitha Pinnagoda (CHU Toulouse), Nicolas Henric (CHU Angers), Olivier Azzis (CHU Rennes), Olivier Rosello (CHU Nice), Poddevin Francois (GHICL), Sara Etienne (CHU Saint Etienne); Philippe Buisson (CHU Amiens Picardie), Sophian Hmila (Hopital Robert Ballanger, Paris);

**Ghana**: Joe-Nat Clegg-Lamptey (Korle Bu Teaching Hospital), Osman Imoro (Baptist Medical Centre), Owusu Emmanuel Abem (Komfo Anokye Teaching Hospital), Paul Wondoh (Upper West Regional Hospital).

**Greece**: Dimitrios Papageorgiou (Naval and Veterans Hospital of Athens), Vasiliki Soulou (Anticancer Hospital of Athens Agios Savvas).

**Guatemala**: Sabrina Asturias (Hospital Herrera Llerandi Amedesgua), Lenin Peña (Hospital General San Juan De Dios).

**India**: Basant Kumar (Sanjay Gandhi Post Graduate Institute of Medical College Lucknow).

**Ireland**: Donal B O’Connor (Tallaght Hospital, Trinity College Dublin).

**Italy**: Alberto Realis Luc (Santa Rita Clinic, Vercelli), Alfio Alessandro Russo (Treviglio Hospital), Andrea Ruzzenente (Azienda Ospedaliera Universitaria Integrata di Verona), Antonio Taddei (Azienda Ospedaliera Universitaria Careggi), Camilla Cona (IOV - Istituto Oncologico Veneto), Corrado Bottini (Sant'Antonio Abate Hospital, Gallarate), Giovanni Pascale (Azienda Ospedaliero-Universitaria di Ferrara), Giuseppe Rotunno (Nicola Giannettasio Hospital, Rossano), Leonardo Solaini (University Of Brescia, Spedali Civili Di Brescia ), Marco Maria Pascale (Fondazione Policlinico Universitario 'Agostino Gemelli' ), Margherita Notarnicola (University Of Bari 'Aldo Moro'), Mario Corbellino (Ospedale Luigi Sacco Milano), Michele Sacco (Federico II University of Naples), Paolo Ubiali (Azienda per L'Assistenza Sanitaria N. 5 'Friuli Occidentale', Pordenone), Roberto Cautiero (Second University Of Naples), Tommaso Bocchetti, (Sant’Andrea Hospital, Sapienza University of Rome), Elena Muzio, (S. Andrea Hospital, Poll-Asl 5, La Spezia); Vania Guglielmo (Policlinico Umberto I, Emergency Surgery Department); Eugenio Morandi (Ospedale di Rho – ASST Rhodense), Patrizio Mao (San Luigi Gonzaga Hospital, Orbassano); Emilia De Luca (Department of Medical and Surgical Sciences, Policlinico Universitario Mater Domini Campus Salvatore Venuta, Catanzaro), Margherita Notarnicola (Azienda Ospedaliero Universitaria Consorziale Policlinico Di Bari).

**Jordan**: Farah Mahmoud Ali (Jordan University Hospital).

**Lithuania**: Justas Žilinskas (Klaipeda Republic), Kestutis Strupas (Vilnius University Hospital), Paulius Kondrotas (Taurage County Hospital), Robertas Baltrunas (Rokiskis District Municipality Hospital); Juozas Kutkevicius (Department of General Surgery, Lithuanian University of Health Sciences), Povilas Ignatavicius (Hospital of Lithuanian University of Health Sciences Kaunas Clinics).

**Malaysia**: Choy Ling Tan (Hospital Sultanah Aminah), Jia Yng Siaw (Hospital Sibu), Sir Young Yam (Penang Medical College); Ling Wilson (Sarawak General Hospital), Mohamed Rezal Abdul Aziz (University Malaya Medical Centre).

**Malta**: John Bondin (Mater Dei Hospital).

**Mexico**: Carmina Diaz Zorrilla (Hospital Espanol De Veracruz).

**Morocco**: Anass Majbar (Centre Hospitalier Ibn Sina Rabat).

**Nigeria**: Danjuma Sale (Barau Dikko Teaching Hospital), Lawal Abdullahi (Kano Aminu), Olabisi Osagie (University of Abuja Teaching Hospital), Omolara Faboya (Lagos Lasuth); Adedeji Fatuga (Lagos Luth), Agboola Taiwo (Babcock University Teaching Hospital), Emeka Nwabuoku (Ahmadu Bello University Teaching Hospital).

**Norway**: Marte Bliksøen (Oslo University Hospital).

**Pakistan**: Zain Ali Khan (Bahawal Victoria Hospital, Bahawalpur).

**Paraguay**: Jazmin Coronel (Hospital de Clínicas, II Cátedra de Clínica Quirúrgica, Universidad Nacional de Asunción)

**Peru**: Cesar Miranda (Hospital Nacional Cayetano Heredia), Idelso Vasquez (Lima Almenara), Luis M. Helguero-Santin (Hospital Regional III Jose Cayetano Heredia – Piura).

**Rwanda**: Jennifer Rickard (Centre Hospitalier Universitaire De Kigali).

**Romania**: Aurel Mironescu (Spitalul Clinic De Copii Brasov).

**Saint Kitts and Nevis**: Adesina Adedeji (Joseph N France Hospital).

**Saudi Arabia**: Saleh Alqahtani (King Fahad General Hospital).

**South Africa**: Max Rath (Groote Schuur Hospital), Michael Van Niekerk (New Somerset Hospital), Modise Zacharia Koto (Dr George Mukhari Academic Hospital); Roel Matos-Puig (Stanger Hospital).

**Sweden**: Leif Israelsson (Sundsvall).

**Switzerland**: Tobias Schuetz (Kantonsspital Olten).

**Turkey**: Mahmut Arif Yuksek (Ondokuz Mayis University), Meric Mericliler (Acibadem University School of Medicine, Atakent Hospital), Mehmet Uluşahin (Karadeniz Technical University Farabi Hospital).

**United Kingdom**: Bernhard Wolf (Raigmore Hospital Inverness), Cameron Fairfield (Royal Infirmary Of Edinburgh), Guo Liang Yong (Perth Royal Infirmary), Katharine Whitehurst (Royal Devon And Exeter), Michael Wilson (Ninewells Hospital And Medical School), Natalie Redgrave (John Radcliffe Hospital, Oxford); Caroluce K Musyoka (Royal Alexandra Hospital), James Olivier (Royal United Hospital Bath), Kathryn Lee (Queen Elizabeth Birmingham), Michael Cox (Royal Derby Hospital), Muhamed M H Farhan-Alanie (Inverclyde Royal Hospital), Rory Callan (North Middlesex University Hospital)

**Zambia**: Chali Chibuye (Ndola Central Hospital)

*GlobalSurg Collaborative protocol translators:*

**Arabic,** Tebian Hassanein Ahmed Ali, Syrine Rekhis, Muna Rommaneh, Oday Halhouli**.**

**Chinese,** Zi Hao Sam**.**

**French,** Lawani Ismaïl**.**

**Greek,** Vasileios Kalles**.**

**Italian,** Francesco Pata, Gabriela Elisa Nita, Federico Coccolini, Luca Ansaloni**.**

**Portuguese,** Thays Brunelli Pugliesi, Gabriel Pardo**.**

**Spanish,** Ruth Blanco

**Appendix S2: Methods**

**Economic evaluation**

This was a model-based cost-effectiveness analysis (CEA) that used published data, clinical data from the PENGUIN pilot study and a cost survey conducted to estimate the perioperative costs of oxygen and SSI treatment among abdominal surgery patients. Separate models were built for each of the countries in the study because unit costs are different across countries. Clinical data were collected from PENGUIN pilot study in India and South Africa (Table S1). The evaluation assessed the costs and outcomes of high fraction of inspired oxygen (FiO_2_) compared to low FiO_2_. All the costs were reported in 2020 US dollars and outcomes were measured as SSI where SSI was defined as an infection of the only skin and subcutaneous tissue of the incision with either purulent drainage, organisms, fever, pain, swelling, heat, clinical or radiology diagnosis or the wound opened spontaneously within 30 days of surgery^13^.

Table S1: Hospitals from which PENGUIN pilot study clinical data were sourced

| **Hospital name** | **Country** | **Hospital type** |
| --- | --- | --- |
| Christian Medical Hospital, Ludhiana | India | Teaching hospital |
| Tata Memorial, Kolkata | India | Cancer hospital and research Centre |
| India Institute of Medical Sciences, Rishkesh | India | Teaching hospital |
| All India Institute of Medical Sciences, Bhubaneswar | India | Teaching hospital |
| Christian Medical College, Vellore | India | Teaching hospital |
| All India Institute of Medical Sciences, Jodhpur | India | Teaching hospital |
| Christian Hospital Chinchpada | India | General hospital |
| Government Medical College and Rajindra Hospital Patiala | India | Teaching hospital |
| Homi Bhaba Cancer Hospital Sangur | India | Cancer hospital |
| Government Medical College & Hospital Chandigarh | India | Teaching hospital |
| Amrita institute Kochi | India | Teaching hospital |
| Tata Memorial Hospital Mumbai | India | Teaching hospital |
| Groote Schuur Hospital | South Africa | Teaching hospital |

Data on SSI incidence were collected from the PENGUIN pilot study questionnaire. The questionnaire was administered by a trained blinded assessor who asked standardised questions to establish whether the patient had an SSI.

Table S2: Unbundled data sourced from the PENGUIN Pilot study

| **Variable** | **No. of patients with the event** | **Total sample** | **Missing outcome** |
| --- | --- | --- | --- |
| In-patient SSI | 49 | 585 | - |
| Unresolved in-patient SSI | 20 | 38 | 11 |
| Re-intervention following in-patient SSI | 10 | 20 | - |
| Post-discharge SSI* | 8 | 444 | 92 |
| Unresolved post-discharge SSI** | 1 | 8 | - |
| Post discharge re-intervention*** | 1 | 436 | - |

Sourced from unbundled/combined data of 585 high and low FiO_2_ patients (558 from India and 27 from South Africa) participating in the PENGUIN pilot study. Post-discharge SSI* stands for SSI occurring after the patient was discharged from the hospital. Unresolved post-discharge SSI** stands for SSI that occurred after hospital discharge and did not resolve. Post discharge re-intervention*** stands for unresolved post discharge SSI that needed re-intervention.

**Cost estimation**

*Sources of costs*

Direct healthcare resource use data were sourced from tertiary and teaching hospitals in Nigeria, India and South Africa that are part of Global Surgery Unit. Responses were received from nine of the 26 teaching and federal medical centres/tertiary hospitals that are part of the Global Surgery Unit in Nigeria. Responses were received from three teaching hospitals out of 21 hospitals that are part of the Global Surgery Unit in India and two tertiary hospitals in South Africa. Clinicians and hub managers from the hospitals completed an online questionnaire designed to capture perioperative and SSI treatment resource usage during abdominal surgery. The questionnaire in this Appendix was piloted in Nigeria before being iteratively improved and disseminated to the other participating centres and countries.

Table S3: Resource use and unit cost questionnaire (Part A: to be completed by an anaesthetist)

| **Oxygen delivery costs and usage** | | | | | | | | | | |
| --- | --- | --- | --- | --- | --- | --- | --- | --- | --- | --- |
| **Hospital Name** | **______________________________________________________** | | | | | | | | | |
| **Resource Use** | | | | | | | | | | |
| Which of the following gases do you administer during surgery?  *(Tick all that apply)* | | | | - Oxygen - Medical air - Nitrous Oxide | | | | | | |
| Breathing system used?  (*Tick all that apply*) | | | | - Circle system - Other circuits | | | | | | |
| Oxygen supply for anaesthesia (*tick all that apply*) | | | | - Oxygen concentrator in operating room - Oxygen cylinder - Pipeline from vacuum insulated evaporator (VIE) - Pipeline from oxygen cylinder manifold | | | | | | |
| Method of administering oxygen/ventilation | | | | - Nasal prong - Non breathing face mask - Laryngeal mask airway (LMA) - Endotracheal tube (ETT) | | | | | | |
| On average, how long does laparotomy procedure take? (minutes) | | | | Indicate time min | | | | | | |
| For high FiO_2,_ what is the average fresh gas flow rate used during surgery? | | | | Fresh gas flow rate (L/min) | | | | | | |
| Mixture to lower FiO_2_?  (*Tick all that apply*) | | | | - Cylinder nitrous oxide - Pipeline nitrous oxide - Cylinder medical air | | | | | | |
| For low FiO_2,_what is the average fresh gas flow rate used during surgery? | | | | Fresh gas flow rate (L/min) | | | | | | |
| What are the average sizes of the cylinders for each of the gas delivery methods? | | | | - Oxygen concentrator (litres) - Oxygen cylinder (litres) - (Pipeline)/ VIE (litres) - (Pipeline)/ manifold cylinders (litres) | | | | | | |
| **Form completed by** | | | | | | | | | | |
| Full print name | | ______________________________________________________ | | | | | | | | |
| Date form completed | | D | D | | M | M | Y | Y | Y | Y |

Table S4: Resource use and unit cost questionnaire (Part B: to be completed by Hub Manager)

| **Unit costs** | | | | | | | | |
| --- | --- | --- | --- | --- | --- | --- | --- | --- |
| How much does it cost to refill oxygen concentrator? *(Indicate zero if there is no cost)* | | | Indicate cost here ___________________ | | | | | |
| How much does it cost to refill the theatre oxygen cylinder? | | | Indicate cost here ___________________ | | | | | |
| How much does it cost to refill the VIE? | | | Indicate cost here ___________________ | | | | | |
| How much does it cost to refill the manifold cylinder? | | | Indicate cost here ___________________ | | | | | |
| What is the average cost of a surgical ward patient (per day)? | | | Indicate cost here ____________________ | | | | | |
| What is the average cost of disposables for in-patient wound dressing change? | | | Indicate cost here ____________________ | | | | | |
| What is the average cost of disposables for out-patient wound dressing change? | | | Indicate cost here ____________________ | | | | | |
| What is the average health worker cost for out-patient wound review (cost of nurse/doctor/community health worker)? | | | Indicate cost here ____________________ | | | | | |
| On average, how many out-patient wound reviews are made per patient? | | | Indicate number here ____________ | | | | | |
| What is the most common empirical antibiotic used for treating surgical site infections? | | | Indicate antibiotic name here ____________ | | | | | |
| On average, how much does the empirical antibiotic cost (overall treatment cost)? | | | Indicate cost here ____________________ | | | | | |
| What is the most frequent used post-operative painkiller? | | | Indicate painkiller name here ____________ | | | | | |
| How much does the most frequent post-operative painkiller cost (overall treatment cost)? | | | Indicate cost here ___________________ | | | | | |
| What is the cost of wound swab cost including analysis (entire treatment cost per patient)? | | | Indicate cost here ___________________ | | | | | |
| What is the average cost of re-intervention/reoperation for wound complication? | | | Indicate cost here ____________________ | | | | | |
| **Form completed by** | | | | | | | | |
| Full print name | ________________________________________________ | | | | | | | |
| Date form completed | D | D | M | M | Y | Y | Y | Y |

To estimate the amount of FiO_2_ gas utilised per patient, the fresh gas flow rate was multiplied by the duration of abdominal surgery and FiO_2_ concentration rate. The cost of refilling manifold or cylinder was divided by the size of the manifold or cylinder to estimate the cost of the gas per litre. In the base-case, the median values of the concentration rates for both arms were used (90% and 28% FiO_2_ concentration rates for high and low FiO_2_ respectively). Then the amount of gas used the patient was multiplied with the cost of gas per litre.

Table S5: Length of hospital stay (LoS)

| **Variable** | **Mean LoS in days (SD)** | **Median LoS in days (IQR)** | **Distribution** | **Source** |
| --- | --- | --- | --- | --- |
| SSI | 16 (6) | 16 (11) | Fixed | PENGUIN pilot trial^9^ |
| No SSI | 8 (4) | 7 (5) | Fixed | PENGUIN pilot trial^9^ |
| Re-intervention | 18 (17) | 15 (21) | Fixed | GlobalSurg Collaborative^16^ |

SD stands for standard deviation. The LoS were sourced from clinical trials data, and it was assumed that LoS are identical between the two arms in the current model.

Table S6: Resource use and unit costs: Nigeria

| **Resource use** | **Frequency** | **Time/cost ($): mean (SD)** | **Cost (Naira)** | **Distribution** | **Source** |
| --- | --- | --- | --- | --- | --- |
| Laparotomy duration: time, minute | 1 | 120.00 | N/A | Fixed | Survey |
| Reintervention | 1 | 133.43 (76.92) | 47,875.00 | Gamma | Survey |
| Swab including analysis | 1 | 16.65 (32.82) | 5938.00 | Gamma | Survey |
| Health worker outpatient wound review | 1 | 18.12 (12.15) | 6500.00 | Gamma | Survey |
| Postoperative painkiller | 1 | 11.43 (13.32) | 4100.00 | Gamma | Survey |
| Postoperative antibiotic | 1 | 40.51 (53.40) | 14,534.00 | Gamma | Survey |
| Outpatient wound dressing change | 1 | 3.38 (2.65) | 1214.00 | Gamma | Survey |
| In-patient wound dressing change | 1 | 3.38 (2.59) | 1213.00 | Gamma | Survey |
| Surgical bed day | 1 | 12.46 (18.30) | 4471.00 | Gamma | Survey |
| Discharge medications | 1 | 8.93 (7.97) | 11,600.00 | Gamma | FALCON^19^ |
| Healthcare revisit cost | 1 | 26.08 (23.28) | N/A | Gamma | Imputed* |
| Purchased medications | 1 | 23.05 (0.05) | N/A | Gamma | Imputed* |
| Oxygen cylinder refill per litre | N/A | 0.01 (0.03) | 5.19 | Gamma | Survey |
| Manifold cylinder refill per litre | N/A | 0.01 (0.02) | 2.74 | Gamma | Survey |
| Low FiO_2_ | 1 | 0.77 (2.54) | 276.49 | Gamma | Survey |
| High FiO_2_ | 1 | 9.37 (28.82) | 3361.00 | Gamma | Survey |

Column 3 reports all resource use values in costs apart from duration of laparotomy which is reported in time (minutes). N/A means not applicable. SD means standard deviation. Survey refers to the survey in Appendix S2 above. $ stands for 2020 US dollars while Naira stands for Nigerian Naira. *Imputed refers to values imputed using the market-basket approach.

Table S7: Resource use and unit costs: India

| **Resource use** | **Frequency** | **Time/cost ($): mean (SD)** | **Cost (Rupee)** | | **Distribution** | **Source** |
| --- | --- | --- | --- | --- | --- | --- |
| Laparotomy duration: time, minute | 1 | 86.67 | N/A | | Fixed | Survey |
| Reintervention | 1 | 142.91 (192.09) | 10,590.00 | | Gamma | Survey |
| Swab including analysis | 1 | 9.67 (9.45) | 716.67 | | Gamma | Survey |
| Health worker outpatient wound review | 2 | 1.42 (1.81) | 105.00 | | Gamma | Survey |
| Postoperative painkiller | 1 | 5.4 (2.94) | 400.00 | | Gamma | Survey |
| Postoperative antibiotic | 1 | 37.34 (61.20) | 2766.67 | Gamma | | Survey |
| Outpatient wound dressing change | 1 | 1.8 (1.4) | 133.33 | | Gamma | Survey |
| In-patient wound dressing change | 1 | 3.26 (0.01) | 241.67 | | Gamma | Survey |
| Surgical bed day | 1 | 8.93 (9.73) | 661.67 | | Gamma | Survey |
| Discharge medications | 1 | 7.85 (9.26) | 582.00 | | Gamma | PENGUIN pilot^9^ |
| Healthcare revisit cost | 1 | 35.01 (49.59) | 2594.00 | | Gamma | PENGUIN pilot^9^ |
| Purchased medications | 1 | 28.62 (49.11) | 2121.00 | | Gamma | PENGUIN pilot^9^ |
| Oxygen cylinder refill per litre | N/A | N/A | N/A | | Gamma | Survey |
| Manifold cylinder refill per litre | N/A | 0.05 (0.05) | 1.17 | | Gamma | Survey |
| Low FiO_2_ | 1 | 0.44 (2.54) | 36.85 | | Gamma | Survey |
| High FiO_2_ | 1 | 4.77 (0.39) | 353.36 | | Gamma | Survey |

Colum 3 reports all resource use values in costs apart from duration of laparotomy which is reported in time (minutes). N/A means not applicable. SD means standard deviation. Survey refers to the survey in Appendix S2. $ stands for 2020 US dollars while Rupee stands for Indian Rupee

Table S8: Resource use and unit costs: South Africa

| **Resource use** | **Frequency** | **Time/cost ($): mean (SD)** | **Cost (Rand)** | **Distribution** | **Source** |
| --- | --- | --- | --- | --- | --- |
| Laparotomy duration: time, min | 1 | 143.00 | N/A | Fixed | Survey |
| Reintervention | 1 | 932.14 (233.03) | 15343.00 | Gamma | Survey |
| Swab including analysis | 1 | 81.00 (4.92) | 81.00 | Gamma | Survey |
| Health worker outpatient wound review | 1 | 23.69 (5.92) | 390.00 | Gamma | Survey |
| Postoperative painkiller | 1 | 6.68 (1.67) | 110.00 | Gamma | Survey |
| Postoperative antibiotic | 1 | 31.03 (7.76) | 510.72 | Gamma | Survey |
| Outpatient wound dressing change | 1 | 0.14 (1.00) | 2.27 | Gamma | Survey |
| In-patient wound dressing change | 1 | 0.14 (0.03) | 2.27 | Gamma | Survey |
| Surgical bed day | 1 | 61.54 (15.39) | 1013.00 | Gamma | Survey |
| Discharge medications | - | - | - | Gamma | Survey |
| Healthcare revisit cost | 1 | 12.36 (21.82) | 197.78 | Gamma | PENGUIN pilot^9^ |
| Purchased medications | 1 | 13.56 (0.05) | 0.10 | Gamma | PENGUIN pilot^9^ |
| Oxygen cylinder refill per litre | N/A | - | - | Gamma | PENGUIN pilot^9^ |
| Manifold cylinder refill per litre | N/A | 0.001 (0.001) | 0.01 | Gamma | Survey |
| Low FiO_2_ | 1 | 1.33 (0.33) | 21.27 | Gamma | Survey |
| High FiO_2_ | 1 | 4.27 (1.07) | 68.38 | Gamma | Survey |

Column 3 reports all resource use values costs apart from duration of laparotomy which is reported in time (min). N/A means not applicable. SD means standard deviation. Survey refers to the survey in Appendix S2. Rands stand for South African Rands while $ stands for 2020 US dollars.

Table S9: Median costs calculated from data used in Table S6-8

|  | **Nigeria** | **India** | **South Africa** |
| --- | --- | --- | --- |
| **Resource use** | **Median costs, $ (IQR)** | **Median costs, $ (IQR)** | **Median costs, $ (IQR)** |
| Re-intervention | 94.76 (101.03) | 67.48 (180.63) | - |
| Swab including analysis | 4.88 (4.53) | 6.75 (9.11) | - |
| Health worker cost for outpatient wound review | 20.90 (16.72) | 1.42 (1.28) | - |
| Postoperative painkiller | 8.36 (11.50) | 4.05 (2.70) | - |
| Postoperative antibiotic | 27.87 (22.30) | 4.05 (53.98) | - |
| Outpatient wound dressing change | 2.79 (2.79) | 1.35 (1.35) | - |
| In-patient wound dressing change | 2.79 (3.14) | 2.70 (3.21) | - |
| Surgical bed day | 8.36 (20.90) | 6.75 (9.55) | - |
| Discharge medications | N/A* | 5.00 (4.00) | - |
| Healthcare revisit cost | N/A* | 11.00 (32.00) | 15.00 (3.00) |
| Purchased medications | N/A* | 13.00 (28.00) | 11.00 (12.00) |
| Oxygen cylinder refill per litre | 0.01 (0.73) | N/A | - |
| Manifold cylinder refill per litre | 0.01 (4.81) | 0.02 (0.01) | - |
| Low FiO_2_ | 0.49 (0.21) | 0.53 (0.38) | - |
| High FiO_2_ | 4.13 (8.68) | 5.85 (4.20) | - |

IQR stands for interquartile range. Most South African data were from published average costs so we could not estimate the median. *Could not estimate the median values as the costs were inputted.

**Market-basket cost imputation approach**

The market-basket approach was used to estimate the cost of healthcare revisit in Nigeria.^18^ Cost of nine items (resource use) available in Nigeria, India, and South Africa were collected from a survey as part of this study: Re-intervention, swab (including analysis), health worker for outpatient wound review, postoperative painkiller, empirical antibiotic, outpatient wound dressing change, in-patient wound dressing change, low FiO_2_ and high FiO_2_. We collected the unit cost of the nine items in the three countries, multiplied each resource use by the associated frequency of use and summed the cost of the nine items to calculate the total cost of the basket in each country. Then, we divided the cost of the basket in Nigeria by the cost of the basket in India and South Africa to obtain the Nigeria-India Index and Nigeria-South Africa Index respectively.

The healthcare revisit cost in India and South Africa sourced from the PENGUIN pilot study were multiplied by the Nigeria-India index and Nigeria-South Africa index respectively. The results from multiplying the Indian and South African healthcare revisit costs and the associated index were added and then divided by 2 to get a weighted average of healthcare revisit cost in Nigeria.

Table S10: Hospitals that provided healthcare resource use data

| **Hospital name** | **Country** | **Type** |
| --- | --- | --- |
| Christian Medical Hospital, Ludhiana | India | Teaching hospital |
| All India Institute of Medical Sciences, Jodhpur | India | Teaching hospital |
| Government Medical College and Rajindra Hospital Patiala | India | Teaching hospital |
| University Teaching Hospital Shika Zaria Nigeria | Nigeria | Teaching hospital |
| Federal medical centre Birnin, Kebbi Nigeria | Nigeria | Tertiary hospital |
| Aminu Kano Teaching Hospital, Kano | Nigeria | Teaching hospital |
| Federal Medical Centre Gusau, Zamfara | Nigeria | Tertiary hospital |
| Barau Dikko University Teaching Hospital, Kaduna | Nigeria | Teaching hospital |
| Federal Medical Centre Katsina | Nigeria | Teaching hospital |
| Obafemi Awolowo University Teaching Hospital, Ile-Ife, Osun state | Nigeria | Teaching hospital |
| [Usmanu Danfodiyo University Teaching Hospital](https://www.uduth.org.ng/)  (UDUTH SOKOTO) | Nigeria | Teaching hospital |
| University College Hospital Ibadan | Nigeria | Teaching hospital |
| Groote Schuur Hospital | South Africa | Teaching hospital |
| University of Western Cape | South Africa | Teaching hospital |

**Sensitivity analysis**

Sensitivity analysis was conducted to test sensitivity of the model results to changes in model parameters inputs. Deterministic sensitivity analysis (DSA) was conducted to test sensitivity of the results to changes in individual model input parameters whilst probabilistic sensitivity analysis (PSA) was conducted to test the robustness of the results to simultaneous changes of multiple parameters in the model

*Deterministic sensitivity analysis*

In the DSA, where necessary the ICER was calculated to show the incremental cost associated with reducing SSIs by one percentage point. Where the results showed dominance of either high or low FiO_2_, (being cheaper and more effective) the ICER was not calculated as dominance means cost-effective in favour of the arm that is cheaper and more effective.^11^

The following DSAs were conducted:

1. Considered inclusion of travel costs to revisit the healthcare facilities (direct non-healthcare costs, defined as other costs beyond the healthcare costs that were incurred to access healthcare).^11^ In this study the non-direct healthcare costs included costs for purchased medication and travel.
2. High FiO_2_ concentration rate was varied from 90% to 80% and 100% while low FiO_2_ was varied from 28% to 21% and 35%_._
3. Considered the lower and upper bounds associated with SSI and reintervention rates, and SSI LoS (Table S11).
4. In the high FiO_2_ arm, threshold analysis was conducted to identify values of SSI LoS and FiO_2_ cost at which changes in the input parameters would change the base case results.

Table S11: Parameter values used in deterministic sensitivity analysis

| **Parameter** | **Base value** | **DSA value** |
| --- | --- | --- |
| Costs included | Non-direct healthcare costs not included | Non-direct healthcare costs included |
| RRR | 12.50% | 5.20% |
|  |  | 19.70% |
| **Low FiO_2_** | | |
| FiO_2_ concentration | 28% | 21.00% |
|  |  | 35.00% |
| SSI probability | 0.08 | 0.06 |
|  |  | 0.11 |
| Reintervention* probability | 0.50 | 0.28 |
|  |  | 0.72 |
| SSI LoS | 15.61 | 9.56 |
|  |  | 21.66 |
| **High FiO_2_** | |  |
| FiO_2_ concentration | 90.00% | 80.00% |
|  |  | 100.00% |
| SSI probability | 0.07 | 0.05 |
|  |  | 0.09 |
| Reintervention* probability | 0.50 | 0.28 |
|  |  | 0.72 |
| SSI LoS | 15.61 | 9.56 |
|  |  | 21.66 |

DSA value means the value that was used in the DSA in place of the base case input parameter value. RRR means relative risk reduction. Re-intervention* stands for re-intervention following in-patient SSI. SSI LoS stands for length of hospital stay for patients who had an SSI.

*Probabilistic sensitivity analysis (PSA)*

Joint uncertainty of multiple changes to the probabilities and costs were assessed using the PSA that was run for 10,000 Monte-Carlo simulations based on the distributions assigned to the parameters. Random values of the costs and probabilities were drawn based on the distributions assigned to the parameters for the 10,000 simulations. The Gamma distribution is restricted to a range from zero to positive infirmity and can be used to characterize uncertainty of costs data.^10^ Beta distribution can be used for proportions because it is restricted to a range from 0 to 1.^10^ As such, costs were assigned a Gamma distribution while probabilities were assigned a Beta distribution.

*Cost-effectiveness acceptability curves*

Uncertainty of the base case results across a range of CETs were assessed using the cost-effectiveness acceptability curves (CEACs).^10^ The net monetary benefit (NMB) approach was used to calculate the probability of high and low FiO_2_ being cost-effective.^11^ Because of lack of specific thresholds for natural units as a measure of outcomes, GDP based CETs were used in all the three countries across a range of CETs. Using the thresholds, an intervention is considered cost-effective if it is less than three times a country’s GDP per capita.^29^

The net monetary benefit was calculated as follows:

Net monetary benefit = CET * difference in outcomes – difference in costs.

**Appendix S3: Results**

**Base case results**

The base case results based on the available evidence on the clinical effectiveness and the minimum important clinical difference (MID) of 12.5% shows that high FiO_2_ is cost-effective as it is cheaper and more effective at reducing SSI compared to low FiO_2_.

**Deterministic sensitivity analysis**

*Nigeria*

When the purchased medication and travel costs were included (DSA I), high FiO_2_ reduced costs by $6.45 (from $6.18 in the base case) and SSIs by 1.05 percentage points. The results were sensitive to changes that were made in favour of low FiO_2_. For example, when the SSI probability in the low FiO_2_ arm was reduced from 0.08 to 0.06, high FiO_2_ was dominated as it increased costs by $25.50 and increased SSIs by 1.20 percentage points (DSA III). The threshold analysis showed that the LoS of patients that had in-patient SSI in the intervention arm would have to increase from 15.61 to 22.38 days or the cost of high FiO_2_ would have to increase from $9.37 to $10.02 for high FiO_2_ not to be cost-effective (DSA IV). The rest of the results have been presented in Table S12.

Table S12: Deterministic sensitivity results (Nigeria)

| **Parameter** | **Base case value** | **DSA value** | **Difference in costs** | **Difference in outcomes** | **ICER/Comment** |
| --- | --- | --- | --- | --- | --- |
| Base case |  |  | -$6.18 | 1.05% | High FiO_2_ dominates |
| Non-direct healthcare costs included | | | -$6.45 | 1.05% | High FiO_2_ dominates |
| RRR | 12.50% | 5.20% | $2.45 | 0.44% | $562.46 |
|  |  | 19.70% | -$14.69 | 1.65% | High FiO_2_ dominates |
| **Low FiO_2_** |  |  |  |  |  |
| FiO_2_ | 28.00% | 21.00% | -$5.99 | 1.05% | High FiO_2_ dominates |
|  |  | 35.00% | -$6.37 | 1.05% | High FiO_2_ dominates |
| SSI | 0.08 | 0.06 | $25.50 | -1.20% | Low FiO_2_ dominates |
|  |  | 0.11 | -$37.86 | 3.29% | High FiO_2_ dominates |
| Reintervention* | 0.50 | 0.28 | $37.90 | 1.05% | $3,620.18 |
|  |  | 0.72 | -$50.26 | 1.05% | High FiO_2_ dominates |
| SSI LoS | 15.61 | 9.56 | $0.14 | 1.05% | $12.92 |
|  |  | 21.66 | -$12.49 | 1.05% | High FiO_2_ dominates |
| **High FiO_2_** | |  |  |  |  |
| FiO_2_ concentration | 0.90 | 0.80 | -$7.22 | 1.05% | High FiO_2_ dominates |
|  |  | 1.00 | -$5.14 | 1.05% | High FiO_2_ dominates |
| SSI | 0.07 | 0.05 | -$35.98 | 3.16% | High FiO_2_ dominates |
|  |  | 0.09 | $23.62 | -1.06% | Low FiO_2_ dominates |
| Reintervention* | 0.50 | 0.28 | -$44.75 | 1.05% | High FiO_2_ dominates |
|  |  | 0.72 | $32.39 | 1.05% | $3,093.88 |
| SSI LoS | 15.61 | 9.56 | -$11.70 | 1.05% | High FiO_2_ dominates |
|  |  | 21.66 | -$0.65 | 1.05% | High FiO_2_ dominates |
| **Threshold analysis** | |  |  |  |  |
| SSI LoS (days) | 15.61 | 22.38 | $0.00 | 1.05% | $0.00 |
| High FiO_2_ cost | $9.37 | $10.02 | $0.00 | 1.05% | $0.00 |

Re-intervention* stands for re-intervention following in-patient SSI. ICER stands for incremental cost-effectiveness ratio. RRR stands for relative risk reduction. SSI LoS stands for length of hospital stay for patients who had an SSI.

*India*

The results were similar to Nigeria as they were sensitive to changes that were made in favour of low FiO_2_ (Table S13). For example, when the probability of getting SSI was increased from 0.07 to 0.09 in the high FiO_2_ arm, high FiO_2_ was dominated as it increased costs by $19.67 and increased SSIs by 1.06 percentage points (DSA IV).

Table S13: Deterministic sensitivity results (India)

| **Parameter** | **Base case value** | **DSA**  **Value** | **Difference in costs** | **Difference in outcomes** | **ICER/Comment** |
| --- | --- | --- | --- | --- | --- |
| Base case | |  | -$10.75 | 1.05% | High FiO_2_ dominates |
| Non-healthcare costs included | | | -$11.22 | 1.05% | High FiO_2_ dominates |
| RRR | 12.50% | 5.20% | -$1.94 | 0.44% | High FiO_2_ dominates |
|  |  | 19.70% | -$19.44 | 1.65% | High FiO_2_ dominates |
| **Low FiO_2_** | | | | | |
| FiO_2_ | 28.00% | 21.00% | -$10.64 | 1.05% | High FiO_2_ dominates |
|  |  | 35.00% | -$10.86 | 1.05% | High FiO_2_ dominates |
| SSI | 0.08 | 0.06 | $21.59 | -1.20% | Low FiO_2_ dominates |
|  |  | 0.11 | -$43.09 | 3.29% | High FiO_2_ dominates |
| Reintervention* | 0.50 | 0.28 | $36.47 | 1.05% | $3,483.03 |
|  |  | 0.72 | -$57.97 | 1.05% | High FiO_2_ dominates |
| SSI LoS | 15.61 | 9.56 | -$6.22 | 1.05% | High FiO_2_ dominates |
|  |  | 21.66 | -$15.27 | 1.05% | High FiO_2_ dominates |
| **High FiO_2_** | | | | | |
| FiO_2_ concentration | 0.90 | 0.80 | -$11.28 | 1.05% | High FiO_2_ dominates |
|  |  | 1.00 | -$10.22 | 1.05% | High FiO_2_ dominates |
| SSI | 0.07 | 0.05 | -$41.17 | 3.16% | High FiO_2_ dominates |
|  |  | 0.09 | $19.67 | -1.06% | Low FiO_2_ dominates |
| Reintervention* | 0.50 | 0.28 | -$52.06 | 1.05% | High FiO_2_ dominates |
|  |  | 0.72 | $30.57 | 1.05% | $2,919.31 |
| SSI LoS | 15.61 | 9.56 | -$14.71 | 1.05% | High FiO_2_ dominates |
|  |  | 21.66 | -$6.79 | 1.05% | High FiO_2_ dominates |
| **Threshold analysis** | |  |  |  |  |
| SSI LoS (days) | 15.61 | 32.04 | $0.00 | 1.05% | $0.00 |
| FiO_2_ cost | $4.77 | $15.52 | $0.00 | 1.05% | $0.00 |

Re-intervention* stands for re-intervention following in-patient SSI. ICER stands for incremental cost-effectiveness ratio. RRR stands for relative risk reduction. SSI LoS stands for length of hospital stay for patients who had an SSI.

*South Africa*

The results were similar to Nigeria and India as there were sensitive to changes in probabilities that were in favour of low FiO_2_. For example, high FiO_2_ was dominated when the probability of getting SSI in the intervention arm was increased from 0.07 to 0.09 as high FiO_2_ increased cost by $95.03 and SSIs by 1.06 percentage points (Table S14).

Table S14: Deterministic sensitivity results (South Africa)

| **Parameter** | **Base case value** | **DSA value** | **Difference in costs** | **Difference in outcomes** | **ICER/Comment** |
| --- | --- | --- | --- | --- | --- |
| Base case | |  | -$93.31 | 1.05% | High FiO_2_ dominates |
| Non-direct healthcare costs included | | | -$93.37 | 1.05% | High FiO_2_ dominates |
| RRR | 12.50% | 5.20% | -$38.78 | 0.44% | High FiO_2_ dominates |
|  |  | 19.70% | -$147.09 | 1.65% | High FiO_2_ dominates |
| **Low FiO_2_** | | | | | |
| FiO_2_ | 28.00% | 21.00% | -$93.30 | 1.05% | High FiO_2_ dominates |
|  |  | 35.00% | -$93.31 | 1.05% | High FiO_2_ dominates |
| SSI | 0.08 | 0.06 | $106.89 | -1.20% | Low FiO_2_ dominates |
|  |  | 0.11 | -$293.51 | 3.29% | High FiO_2_ dominates |
| Reintervention* | 0.50 | 0.28 | $214.66 | 1.05% | $20,502.30 |
|  |  | 0.72 | -$401.27 | 1.05% | High FiO_2_ dominates |
| SSI LoS | 15.61 | 9.56 | -$62.12 | 1.05% | High FiO_2_ dominates |
|  |  | 21.66 | -$124.49 | 1.05% | High FiO_2_ dominates |
| **High FiO_2_** | | | | | |
| FiO_2_ concentration | 0.90 | 0.80 | -$93.32 | 1.05% | High FiO_2_ dominates |
|  |  | 1.00 | -$93.30 | 1.05% | High FiO_2_ dominates |
| SSI | 0.07 | 0.05 | -$281.64 | 3.16% | High FiO_2_ dominates |
|  |  | 0.09 | $95.03 | -1.06% | Low FiO_2_ dominates |
| Reintervention* | 0.50 | 0.28 | -$362.78 | 1.05% | High FiO_2_ dominates |
|  |  | 0.72 | $176.16 | 1.05% | $16,825.55 |
| SSI LoS | 15.61 | 9.56 | -$120.59 | 1.05% | High FiO_2_ dominates |
|  |  | 21.66 | -$66.02 | 1.05% | High FiO_2_ dominates |
| **Threshold analysis** | | | | | |
| SSI LoS (days) | 15.61 | 36.30 | $0.00 | 1.05% | $0.00 |
| High FiO_2_ cost | $0.09 | $93.40 | $0.00 | 1.05% | $0.00 |

Re-intervention* stands for re-intervention following in-patient SSI. ICER stands for incremental cost-effectiveness ratio. RRR stands for relative risk reduction. SSI LoS stands for length of hospital stay for patients who had an SSI.
