# Supplementary material for: Exploring the cost-effectiveness of high versus low perioperative fraction of inspired oxygen in the prevention of surgical site infections among abdominal surgery patients in three low- and middle-income countries
Source: BJA Open. 2023 Jul 15;7:100207. doi: 10.1016/j.bjao.2023.100207 (PMC10457493; doi:10.1016/j.bjao.2023.100207)
Supplement: Multimedia component 2 [file mmc2.docx]

Completed CHEERS 2022 checklist

| **Section/topic** | **No** | **Guidance for reporting** | **Reported in section** |
| --- | --- | --- | --- |
| **Title** |  |  |  |
| Title | 1 | Identify the study as an economic evaluation and specify the interventions being compared | Title page: Page 1 |
| **Abstract** |  |  |  |
| Abstract | 2 | Provide a structured summary that highlights context, key methods, results, and alternative analyses | Abstract: Page 2 |
| **Introduction** |  |  |  |
| Background and objectives | 3 | Give the context for the study, the study question, and its practical relevance for decision making in policy or practice | Introduction: Page 3 |
| **Methods** |  |  |  |
| Health economic analysis plan | 4 | Indicate whether a health economic analysis plan was developed and where available | Not developed |
| Study population | 5 | Describe characteristics of the study population (such as age range, demographics, socioeconomic, or clinical characteristics) | Ethical statement: Page 4 |
| Setting and location | 6 | Provide relevant contextual information that may influence findings | Effect size calculation: Page 5 |
| Comparators | 7 | Describe the interventions or strategies being compared and why chosen | Introduction: Page 3 |
| Perspective | 8 | State the perspective(s) adopted by the study and why chosen | Study perspective: Page 4 |
| Time horizon | 9 | State the time horizon for the study and why appropriate | Model timeframe: Page 5 |
| Discount rate | 10 | Report the discount rate(s) and reason chosen | Outcome measure: Page 6&7 |
| Selection of outcomes | 11 | Describe what outcomes were used as the measure(s) of benefit(s) and harm(s) | Outcome measure: Page 6&7 |
| Measurement of outcomes | 12 | Describe how outcomes used to capture benefit(s) and harm(s) were measured | Outcome measure: Page 6&7 |
| Valuation of outcomes | 13 | Describe the population and methods used to measure and value outcomes | Outcome measure: Page 6&7 |
| Measurement and valuation of resources and costs | 14 | Describe how costs were valued | Resource use and unit costs: Page 6 |
| Currency, price date, and conversion | 15 | Report the dates of the estimated resource quantities and unit costs, plus the currency and year of conversion | Resource Use and Unit costs: Page 6 |
| Rationale and description of model | 16 | If modelling is used, describe in detail and why used. Report if the model is publicly available and where it can be accessed | Type of model: Page 4 |
| Analytics and assumptions | 17 | Describe any methods for analysing or statistically transforming data, any extrapolation methods, and approaches for validating any model used | Statistical analysis and sensitivity analysis: Page 7 |
| Characterizing heterogeneity | 18 | Describe any methods used for estimating how the results of the study vary for subgroups | Study perspective: Page 4 & Resource Use and Unit costs: Page 6 |
| Characterizing distributional effects | 19 | Describe how impacts are distributed across different individuals or adjustments made to reflect priority populations | Study perspective: Page 4 |
| Characterizing uncertainty | 20 | Describe methods to characterise any sources of uncertainty in the analysis | Sensitivity analysis: Page 7 |
| Approach to engagement with patients and others affected by the study | 21 | Describe any approaches to engage patients or service recipients, the general public, communities, or stakeholders (such as clinicians or payers) in the design of the study | N/A* |
| **Results** |  |  |  |
| Study parameters | 22 | Report all analytic inputs (such as values, ranges, references) including uncertainty or distributional assumptions | Model probabilities: Page 5, Length of hospital stay: page 6, resource use and unit costs: page 6 |
| Summary of main results | 23 | Report the mean values for the main categories of costs and outcomes of interest and summarise them in the most appropriate overall measure | Base case results: Page 6 |
| Effect of uncertainty | 24 | Describe how uncertainty about analytic judgments, inputs, or projections affect findings. Report the effect of choice of discount rate and time horizon, if applicable | Results- deterministic sensitivity analysis: Page 8 |
| Effect of engagement with patients and others affected by the study | 25 | Report on any difference patient/service recipient, general public, community, or stakeholder involvement made to the approach or findings of the study | N/A* |
| **Discussion** |  |  |  |
| Study findings, limitations, generalizability, and current knowledge | 26 | Report key findings, limitations, ethical or equity considerations not captured, and how these could affect patients, policy, or practice | Discussion: Page 10-11 |
| **Other relevant information** | |  |  |
| Source of funding | 27 | Describe how the study was funded and any role of the funder in the identification, design, conduct, and reporting of the analysis | Funding: Page 14 |
| Conflicts of interest | 28 | Report authors conflicts of interest according to journal or International Committee of Medical Journal Editors requirements | Conflict of interests: Page 14 |

Source of the checklist: <https://www.bmj.com/content/376/bmj-2021-067975>.

*N/A stands for not applicable as this was a model-based evaluation
